# Direct 2,3-*O*-Isopropylidenation of α-d-Mannopyranosides and the Preparation of 3,6-Branched Mannose Trisaccharides

**DOI:** 10.3390/molecules19056683

**Published:** 2014-05-22

**Authors:** Rui Jiang, Guanghui Zong, Xiaomei Liang, Shuhui Jin, Jianjun Zhang, Daoquan Wang

**Affiliations:** Department of Applied Chemistry, China Agricultural University, Beijing 100193, China

**Keywords:** 2,3-*O*-isopropylidenation, α-d-mannosides, trisaccharide

## Abstract

A highly efficient, regioselective method for the direct 2,3-*O*-isopropylidenation of α-d-mannopyranosides is reported. Treatment of various α-d-mannopyranosides with 0.12 equiv of the TsOH·H_2_O and 2-methoxypropene at 70 °C gave 2,3-*O*-isopropylidene-α-d-mannopyranosides directly in 80%~90% yields. Based on this method, a 3,6-branched α-d-mannosyl trisaccharide was prepared in 50.4% total yield using *p*-nitrophenyl 2,3-*O*-isopropylidene-α-d-mannopyranoside as the starting material.

## 1. Introduction

Many carbohydrates isolated from natural products were found to be involved in a wide range of biological processes. Further investigation of the structure-activity relationships of these carbohydrates normally is often restricted, since the isolated compounds can’t meet the purity and quantity needs of pharmacologists. Thus, the synthesis of structurally complex carbohydrates via chemical methods has become very important, and various kinds of suitably functionalized monosaccharide building blocks are basic requirements in the synthesis of oligosaccharides [1,2,3,4].

The isopropylidene moiety is one of the frequently used protecting groups in oligosaccharide synthesis for the temporary protection of hydroxyl groups. 2,3-*O*-Isopropylidene-α-d-manno-pyranosides are important building blocks in the synthesis of mannose-containing derivatives [5,6,7], and many methods have been reported for the preparation of these compounds. In 1977, Evans [8] disclosed that by the reaction of methyl α-d-mannopyranoside with 2,2-dimethoxypropane in the presence of sulfuric acid for 48 h, methyl 2,3-*O*-isopropylidene-α-d-mannopyranoside was obtained in 56% yield. Obviously, this reaction is time-consuming and the yield is only barely acceptable. In most cases, 2,3-*O*-isopropylidene-α-d-mannopyranosides are prepared in two steps from α-d-manno- pyranosides: 2,3:4,6-di-*O*-isopropylidenation of mannopyranosides followed by selectively removal of 4,6-*O*-isopropylidene groups in the presence of acids. The reported acids including aq. HCl [9], 60% aq. AcOH [10], aq. H2SO_4_ [11], Zn(NO_3_)_2_·6H_2_O [12], BiCl_3_ [13]. To better control the selectivity and efficiency of the hydrolysis, Dowex H^+^ ion-exchange resin [14], FeCl_3_·6H_2_O on silica [15], NaHSO_4_ on silica [16] and HClO_4_ on silica [17] were also used in some cases. Nevertheless, many of these methods suffer from the use of corrosive materials, rigorous reaction conditions and incompatibility with various other protecting groups. Some of the acids used in the reaction are strongly acidic and the selectivity was compromised, leading to decreased yields of the desired products. Here we wish to report a direct and regioselective 2,3-*O*-isopropylidenation of α-d-mannopyranosides method, and its application in the synthesis of mannose oligosaccharide exemplified by the efficient preparation of a 3,6-branched α-d-mannosyl trisaccharide.

## 2. Results and Discussion

**Scheme 1:**
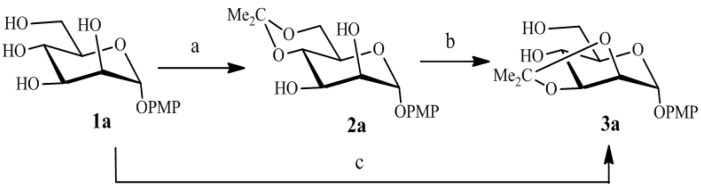
Synthesis of **2a** and **3a**.

As shown in Scheme 1, reaction of the known *p*-methoxyphenyl α-d-mannopyranoside [18] (**1a**, 1 mmol) with 2-methoxypropene (1.05 mmol) in anhydrous *N*,*N*-dimethylformamide containing a trace of *p*-toluenesulfonic acid (0.02 mmol) at room temperature occurs preferentially at the primary hydroxyl group, to give *p*-methoxyphenyl 4,6-*O*-isopropylidene-α-d-mannopyranoside (**2a**) [19]. The reaction was terminated at the end of the reaction (2 h) by addition of triethylamine to neutralize the *p*-toluene-sulfonic acid. Incidentally, we discovered that after extension of the reaction time to 10 h or more without neutralizing the *p*-toluenesulfonic acid, a new by-product **3a** was formed in 20%~30% yields. The by-product **3a** was purified and it was confirmed to be *p*-methoxyphenyl 2,3-*O*-isopropylidene-α-d-mannopyranoside, judging from its ^1^H-NMR and ^13^C-NMR spectrum identical to the published ones [5]. Obviously, rearrangement of the *p*-methoxyphenyl 4,6-*O*-isopropylidene-α-d-mannopyranoside to *p*-methoxyphenyl 2,3-*O*-isopropylidene-α-d-mannopyranoside took place during the course of the reaction. It is important to note that, after a series of experiments, the efficiency of the rearrangement was found to depend on the amount of acid added and the reaction temperature, and the isolated yields of **3a** were greatly improved under the optimized reaction conditions, and finally, reaction of compound **1a** with 2-methoxypropene (1.05 equiv) in anhydrous *N*,*N*-dimethylformamide containing 0.1 equiv of *p*-toluene-sulfonic acid at 70 °C for 4 h, gave **3a** in 93% yield.

*Reagents and Conditions*: (**a**) 2-methoxypropene, TsOH·H_2_O, DMF, r.t., 2 h, 94%; (**b**) r.t., 10 h, 28%; (**c**) 2-methoxypropene, TsOH·H_2_O, DMF, r.t. then 70 °C, 4 h, 93%.

A number of suitably functionalized mannose intermediates **1b** [20], **1c** [6], **1d** [7], **1e** [21], **1f** [22] and **1g** [23] were prepared from the commercially available d-mannose, using earlier reported reaction conditions. With the optimized reaction conditions in hand, α-d-mannopyranosides **1b**~**1f** were used as substrates to investigate the general applicability of the method for the direct synthesis of 2,3-*O*-isopropylidene-α-d-mannopyranosides **3b**~**3f**. Though the reaction conditions are slightly different for each substrate, we were delighted to find that good to excellent yields (85%~93%) of the corresponding 2,3-*O*-isopropylidene-α-d-mannopyranoside derivatives were obtained in all cases, and the structure were confirmed by their NMR data (Table 1).

**Table 1:** Synthesis of 2,3-*O*-isopropylidene-α-d-mannopyranosides derivatives. 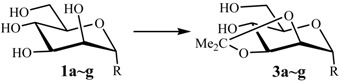

Entry	Reaction	R	TsOH (equiv)	T(°C)	Time(h)	Yield (%)	Ref. ^*^
1	**1a** 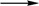 **3a**	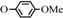	0.1	70	4	93	[5]
2	**1b** 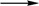 **3b**	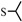	0.3	50	1.5	89	-
3	**1c** 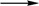 **3c**	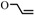	0.1	70	2	91	[6]
4	**1d** 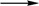 **3d**	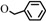	0.3	70	3.5	92	[7]
5	**1e** 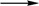 **3e**	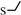	0.5	50	4.5	85	[21]
6	**1f** 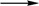 **3f**	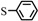	0.1	70	2	88	[22]
7	**1g** 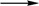 **3g**	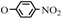	0.3	70	2	90	[23]

* Refs. of known products.

**Scheme 2:**
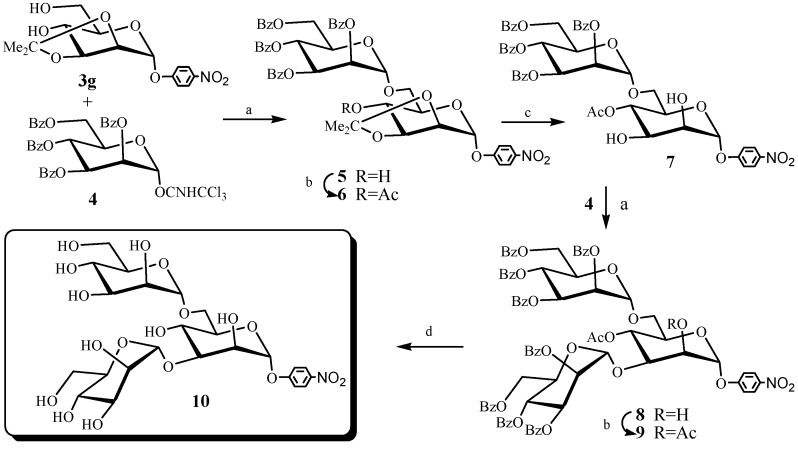
Synthesis of 3,6-branched α-d-mannose trisaccharide **10**.

The above synthesized 2,3-*O*-isopropylidene-α-d-mannopyranosides are useful building blocks for the preparation of mannose-containing oligosaccharides and glycoconjugates. As an example, a 3,6-branched mannose trisaccharide (10, Scheme 2), the core structure of *N*-linked glycan mannose oligosaccharides, was synthesized efficiently. *N*-linked glycan mannose oligosaccharides play a vital role in fundamental biological processes including cell differentiation, malignant transformation, human CD2 adhesion function and HIV infection, and the 3,6-branched mannosyl trisaccharide is reported to be the much better ligand of mannose-specific binding proteins than mono- and linear oligomannosides [24,25,26,27]. Due to their biological importance, 3,6-branched mannosides have become the synthetic focus of many research groups [28,29,30,31] and the development of new synthetic strategies for their efficient construction is of continue interest.

Several synthetic methods have already been reported for 3,6-branched mannose trisaccharides [32,33,34,35,36,37,38,39,40,41]. In 1991, Kaur and Hindsgaul [34] revealed a fifteen-step method for the synthesis of 3,6-branched trisaccharide from commercial available starting materials, but many of these steps involved time-consuming and delicate protecting group manipulations to synthesize 2,4-diprotected mannosides for the subsequent glycosylation. Later, Hindsgaul [35] and Bencomo [37] reported a general approach based on random glycosylation for octyl- and 5-azido-3-oxapentyl-α-d-mannopyranosides, respectively. They used acetobromosugars as the glycosyl donor and mercury (II) cyanide and mercury (III) bromide as the catalysts. In 2000, Kobayashi [38] examined a similar but more convenient glycosylation of *p*-nitrophenyl a-d-mannopyranoside using per-*O*-acetyl-a-d-mannopyranosyl imidate as the donor. The reaction gave a desired 3,6-branched trisaccharide in 42% yield as the major product, together with di-, tri- and tetrasaccharide byproducts. Orthoester chemistry is an efficient method for the synthesis of these 3,6-branched trisaccharides [32,36,40,41,42]. Arnarp and Lonngren [42] described in 1978 a slightly more circuitous route that starts with stannylated benzyl a-d-mannopyranoside, the 3,6-branched mannotrioside was obtained in good yield via a protection-deprotection sequence that circumvents the orthoester rearrangement. In 1993, Oscarson [36], and in 2005, Backinowsky [40] and Mukhopadhyay [41] used orthoester chemistry to provide direct access to a 2,4-diprotected mannoside acceptor for the subsequent glycosylation with a mannoside donor. This approach was more convenient and efficient and the desired 3,6-branched mannotrioside was obtained in satisfactory yield. In this paper we describe a linear synthesis of the target 3,6-branched mannotrioside (**10**) using *p*-nitrophenyl 2,3-*O*-isopropylidene-α-d-mannopyranoside (**3g**) as the mannoside acceptor and 2,3,4,6-tetra-*O*-benzoyl-α-d-mannopyranoside trichloroacetimidate (**4**) as the donor. The 3,6-branched α-d-mannosyl trisaccharide (**10**) was obtained in 50.4% total yield (Scheme 2).

*Reagents and conditions*: (**a**) TMSOTf, CH_2_Cl_2_, −15 °C to rt, 2 h, 92% for **5**, 86% for **8**; (**b**) Py, Ac_2_O, 96% for **6**, 95% for **9**; (**c**) 70% AcOH, 70 °C, 2 h, 91%; (**d**) satd NH_3_-MeOH, rt, 120 h, 73%.

## 3. Experimental Section

### 3.1. General Methods

Optical rotations were determined with a Perkin–Elmer model 241-MC automatic polarimeter for soln in a 1-dm, jacketed cell. ^1^H and ^13^C-NMR spectra were recorded with Bruker DPX300 and Bruker AVANCE600 spectrometers in CDCl_3_ or D_2_O solns. Internal references: TMS (δ 0.000 ppm for ^1^H), CDCl_3_ (δ 77.00 ppm for ^13^C), HOD (δ 4.700 for ^1^H). High-resolution mass spectra (HRMS) was performed by the Peking University. Thin-layer chromatography (TLC) was performed on silica gel HF with detection by charring with 30% (v/v) H_2_SO_4_ in MeOH or by UV detection. Column chromatography was conducted by elution of a column of silica gel (200–300 mesh) with EtOAc/petroleum ether (bp 60–90 °C) as the eluent. Solns were concd at a temperature < 60 °C under diminished pressure.

### 3.2. Chemical Synthesis: Representative Procedure for the Synthesis of 2,3-O-Isopropylidene-α-d-mannopyranosides ***3a**~**3f***

To a solution of compound **1a**~**1g** (2 mmol) in anhydrous DMF (20 mL) was added TsOH·H_2_O (0.2~1 mmol) and 2-methoxypropene (0.2 mL, 2.1 mmol) under N_2_ atmosphere. The mixture was stirred at rt for 1 h and then for another 1~4 h at 50~70 °C, at the end of which time TLC (EtOAc) indicated that the reaction was complete. The reaction mixture was neutralized with Et_3_N, and then concentrated under reduced pressure to remove DMF, the residue was dissolved in CH_2_Cl_2_ (50 mL), and washed with water (20 mL), then the organic phase was dried over Na_2_SO_4_. Evaporation and purification by flash column chromatography afforded compounds **3a**~**3g**.

*p-Methoxyphenyl 2,3-O-isopropylidene-α-D-mannopyranoside* (**3a**): (0.6 g, 93%). 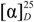 +69.7° (*c* 1.0 CHCl_3_). ^1^H-NMR (300 MHz, CDCl_3_) δ 7.00–6.82 (2m, 4H, Ar-*H* ), 5.67 (s, 1H, *H-*1), 4.38 (d, *J*_2,3_ = 5.7 Hz, 1H, *H-*2), 4.32 (m, 1H, *H*-3), 3.84–3.78 (m, 7H, *H-*4*, H-*5*, H-*6, *O*C*H*_3_), 2.82 (d, *J* = 3.9 Hz, 1H, 4-O*H*), 2.04 (m, 1H, 6-O*H*), 1.56, 1.41 (2s, 6H, *Me*_2_C). ^13^C-NMR (75 MHz, CDCl_3_): δ 155.1, 149.7, 117.8, 114.6, 109.8, 96.4, 78.5, 75.6, 70.2, 69.0, 61.8, 55.5, 27.9, 26.1. HRMS for C_16_H_26_NO_7_ (M+NH_4_)^+^ 344.17038. Found: 344.17035.

*Isopropylthio 2,3-O-isopropylidene-α-d-mannopyranoside* (**3b**): (0.49 g, 89%). 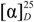 +128.7° (*c* 1.0 CHCl_3_). ^1^H-NMR (300 MHz, CDCl_3_) δ 5.63 (s, 1H, *H*-1), 4.18 (d, *J*_2,3_ = 5.5 Hz, 1H, *H-2*), 4.11 (m, 1H, *H-3*), 4.01 (m, 1H, *H*-5), 3.88–3.77 (m, 3H, *H*-4, *H*-6), 3.10 (m, 1H, SC*H*(CH3)2), 2.89 (d, *J* = 4.0 Hz, 1H, O*H*), 2.16 (m, 1H, O*H*), 1.54, 1.36 (2s, 6H, *Me*2C). 1.35 (d, *J* = 4.8 Hz, 3H, CH(C*H*3)2), 1.30 (d, *J* = 6.9 Hz, 3H, CH(C*H*3)2). HRMS for C_12_H_26_SNO_5_ (M+NH_4_)^+^ 296.15262. Found: 296.15259.

*Allyl 2,3-O-isopropylidene-α-D-mannopyranoside* (**3c**): (0.47 g, 91%). 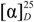 +68.2° (*c* 1.0 CHCl_3_). ^1^H-NMR (300 MHz, CDCl_3_) δ 5.96–5.85 (m, 1H, OCH_2_C*H*CH_2_), 5.33–5.19 (m, 2H, C*H*_2_CHCH_2_O), 4.89 (d, *J*_1,2_ = 1.2 Hz, 1H, *H*-1), 4.21–3.68 (m, 8H, *H*-2, *H*-3, *H*-4, *H*-5, *H*-6, CH_2_CH*CH_2_*O), 2.63–2.57 (m, 2H, 2O*H*), 1.53,1.43 (2s, 6H, *Me_2_C*). HRMS for C_12_H_21_O_6_ (M+H)^+^ 261.13326. Found: 261.13321. 

*Benzyl 2,3-O-isopropylidene-α-d-mannopyranoside* (**3d**): (0.57 g, 92%). 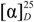 +77.0° (*c* 1.0 CHCl_3_). ^1^H-NMR (300 MHz, CDCl_3_) δ 7.40–7.26 (m, 5H, Ar*H*), 5.12 (s, 1H, *H-*1), 4.74 (d, *J* = 11.7 Hz, 1H, OC*H*_2_Ph), 4.54 (d, *J* = 11.7 Hz, 1H, OC*H*_2_Ph), 4.20–4.16 (m, 2H), 3.85–3.83 (m, 2H), 3.78–3.66 (m, 2H), 3.30–2.88 (s, 1H, O*H*), 2.35 (s, 1H, O*H*), 1.52, 1.34 (2s, 6H, *Me_2_C*). HRMS for C_16_H_26_NO_6_ (M+NH_4_)^+^ 328.17546. Found: 328.17548.

*Ethyl 2,3-O-isopropylidene-1-thio-α-d-mannopyranoside* (**3e**): (0.44 g, 85%). 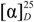 +136.8° (*c* 1.0 CHCl_3_). ^1^H-NMR (300 MHz, CDCl_3_) δ 5.58 (s, 1H, *H-*1), 4.19 (m, 1H, *H*-2), 4.14 (m, 1H, *H*-3), 3.99–3.93 (m, 1H, *H*-5), 3.88–3.77 (m, 3H, *H*-4, *H*-6), 2.74–2.49 (m, 3H, SC*H*_2_, O*H*), 2.07 (t, *J* = 6.5 Hz, 1H, O*H*), 1.54 (s, 3H, *Me*_2_*C*), 1.40–1.25 (m, 6H, SCH_2_C*H*_3_, *Me*_2_*C*). HRMS for C_11_H_24_SNO_5 _(M+NH_4_)^+^ 282.13697. Found: 282.13696.

*Phenyl 2,3-O-isopropylidene-1-thio-α-d-mannopyranoside* (**3f**): (0.55 g, 88%). 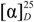 +197.8° (*c* 1.0 CHCl_3_). ^1^H-NMR (300 MHz, CDCl_3_) δ 7.50–7.26 (m, 5H, Ar*H*), 5.81 (s, 1H, *H-*1), 4.35 (d, *J*_2,3_ = 5.5 Hz, 1H, *H*-2), 4.18 (dd, *J*_2,3_ = 5.5 Hz, *J*_3,4_ = 7.5 Hz, 1H, *H*-3), 4.08–4.02 (m, 1H, *H*-5), 3.83–3.70 (m, *H*-4, *H*-6), 2.92 (d, *J* = 4.0 Hz, O*H*), 1.95 (t, 1H, *J* = 6.3 Hz, O*H*), 1.54,1.38 (2s, 6H, *Me_2_C*). HRMS for C_15_H_24_SNO_5_ (M+NH_4_)^+^ 330.13697. Found: 330.13699.

*p*-*Nitrophenyl 2,3-O-isopropylidene-α-d-mannopyranoside* (**3g**): (0.61 g, 90%). 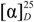 +94.8° (*c* 1.0 CHCl_3_). ^1^H-NMR (300 MHz, CDCl3) δ ppm 8.25–8.20 (m, 2H, Ar-*H*), 7.18–7.13 (m, 2H, Ar-*H*), 5.90 (s, 1H, H-1), 4.38 (d, *J*_2,3_ = 5.7 Hz, 1H, *H*-2), 4.32 (dd, *J*_2,3_ = 5.7 Hz, *J*_3,4_ = 7.3 Hz, 1H, *H*-3), 3.88–3.78 (m, 3H, H-4, 2 × H-6), 3.67–3.62 (m, 1H, H-5), 2.96 (d, 1H, *J* = 4.0 Hz, O*H*), 2.05 (d, 1H, *J* = 3.6 Hz, O*H*), 1.58, 1.42 (2s, 6H, 2 × C-C*H*_3_). HRMS for C_15_H_23_NO_8 _(M+NH_4_)^+^ 359.1449. Found: 359.14481.

*4-Nitrophenyl 2,3,4,6-tetra-O-benzoyl-α-d-mannopyranosyl-(1→6)-2,3-O-isopropylidene-α-d-mannopyranoside* (**5**): To a cooled (−15 °C) solution of **3g** (1.5 g, 4.4 mmol) and **4** (3.4 g, 4.6 mmol) in anhydrous, redistilled CH_2_Cl_2_ (80 mL) was added 4 Å molecular sieves (2 g) and the mixture was stirred under a N_2_ atmosphere for 30 min. Then TMSOTf (16 μL 0.09 mmol, diluted with 10 mL redistilled CH_2_Cl_2_) was added to the mixture dropwise. The reaction mixture was stirred for another 2 h, during which time the mixture was allowed to gradually warm to ambient temperature. TLC (petroleum ether–EtOAc 2:1) indicated that the reaction was complete. Then the reaction mixture was quenched with Et_3_N (2 drops) and filtrated. The filtrate was evaporated *in vacuo* to give a residue, which was purified by silica gel column chromatography (petroleum ether–EtOAc 3:1) to give disaccharide **5** (3.7g, 92%) as a white foam. 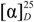 −62.6° (*c* 0.5 CHCl_3_). ^1^H-NMR (300 MHz, CDCl_3_): δ 8.31–7.23 (m, 24H, Bz-*H*, Ar-*H*), 6.07 (t, *J*_3,4_ = *J*_4,5_ = 10.0 Hz, 1H, H-4'), 5.94 (s, 1H, H-1'), 5.74 (dd, *J*_2,3_ = 3.3 Hz, *J*_3,4_ = 10.0 Hz, 1H, H-3'), 5.56 (dd, *J*_1,2_ = 1.7 Hz, *J*_2,3_ = 3.3 Hz, 1H, H-2'), 5.10 (d, 1H, *J*_1,2_ = 1.7 Hz, H-1), 4.74–4.68 (m, 1H), 4.50–4.41 (m, 3H), 4.34 (m, 1H, H-4), 4.01 (m, 1H), 3.91–3.82 (m, 3H), 2.68 (s, 1H, O*H*), 1.60, 1.43 (2s, 6H, 2 × C-C*H*_3_). ^13^C-NMR (75 MHz, CDCl_3_): δ 166.2, 165.6, 165.2(2) (4 × *C*OPh), 160.6 (*C*NO_2_), 142.9, 133.4, 133.1, 133.0, 129.8, 129.7, 129.6, 129.2, 129.0, 128.9, 128.5, 128.4, 128.3, 128.2, 126.0, 116.2, 110.3, 97.6, 95.7 (2 × C-1), 78.5, 75.3, 70.3, 70.0, 69.6, 69.2, 68.9, 67.1, 66.7, 62.9, 28.0, 26.3 (2 × C-*C*H_3_). HRMS for C_49_H_49_N_2_O_17_ (M+NH_4_)^+^ 937.30257. Found: 937.30133.

*4-Nitrophenyl 2,3,4,6-tetra-O-benzoyl-α-d-mannopyranosyl-(1→6)-4-O-acetyl-2,3-O-isopropylidene-α-d-mannopyranoside* (**6**): To a solution of **5** (3.6 g, 4 mmol) in pyridine (30 mL) was added Ac_2_O (3.7 mL, 40 mmol). The reaction mixture was stirred at rt for 12 h, at the end of which time TLC (petroleum ether–EtOAc 2:1) indicated that the reaction was complete. The reaction mixture was concentrated, and then the residue was purified by flash column chromatography on a silica gel column (petroleum ether–EtOAc 3:1) to give compound **6** (3.6 g, 96%) as a foamy solid. 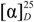 −53.3° (*c* 0.3 CHCl_3_). ^1^H-NMR (300 MHz, CDCl_3_): δ 8.29 (d, *J* = 9.1 Hz, 2H, Bz-*H*), 8.10, 7.80 (2d, *J* = 7.4 Hz, 4H, C_6_*H*_4_NO_2_ ), 7.58–7.24 (m, 14H, Bz-*H* ), 6.05 (t, *J*_3,4_ *= J*_4,5_ = 10.0 Hz, 1H, H-4'), 5.95 (s, 1H, H-1'), 5.65 (dd, *J*_2,3_ = 3.3 Hz, *J*_3,4_ = 10.0 Hz, 1H, H-3'), 5.46 (m, 1H, H-2'), 5.10 (t, *J*_3,4_ = *J*_4,5_ = 10.3 Hz, 1H, H-4), 5.01 (s, 1H, H-1), 4.73 (d, *J*_3,4_ = 10.3 Hz, 1H, H-3), 4.49–4.40 (m, 4H), 4.05–3.88 (m, 2H), 3.53 (m, 1H), 2.21 (s, 3H, C*H*_3_CO), 1.60, 1.42 (2s, 6H, 2 × C-C*H*_3_). ^13^C-NMR (75 MHz, CDCl_3_): δ 169.7 (*C*OCH_3_), 166.1, 165.6, 165.2, 165.1 (4 × *C*OPh), 160.4(*C*NO_2_), 143.1, 133.4, 133.1, 133.0, 129.8, 129.7, 129.6, 129.1, 129.0, 128.9, 128.5, 128.4, 128.2, 126.0, 116.1, 110.6, 97.3, 95.4 (2 × C-1), 77.1, 75.4, 75.1, 70.2, 69.5, 69.3, 69.0, 68.3, 66.9, 66.8, 62.9, 27.5, 26.3(2 × C-*C*H_3_), 20.8 (CO*C*H_3_). HRMS for C_51_H_51_N_2_O_18_ (M+NH_4_)^+^ 979.31314. Found: 979.31201.

*4-Nitrophenyl 2,3,4,6-tetra-O-benzoyl-α-d-mannopyranosyl-(1→6)-4-O-acetyl-α-d-mannopyranoside* (**7**): Compound **6** (3.5 g, 3.6 mmol) was dissolved in 70% AcOH (60 mL) and stirred for 2 h at 70 °C, at the end of which time TLC (petroleum ether–EtOAc 1:1) indicated completion of the reaction. The mixture was concd. under diminished pressure and then coevaporated with toluene (3 × 10 mL). The residue was passed through a short silica-gel column with petroleum ether–EtOAc 3:1 as the eluent to give **7** (3.1 g, 91%) as a white solid. 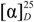 –14.4° (*c* 1.0 CHCl_3_). ^1^H-NMR (300 MHz, CDCl_3_): δ 8.31-7.23 (m, 24H, Bz-*H*, Ar-*H*), 6.05 (t, *J*_3,4_ = *J*_4,5_ = 10.1Hz, 1H, H-4'), 5.81 (d, *J*_2,3_= 3.2 Hz, *J*_3,4_ = 10.1 Hz, 1H, H-3'), 5.77 (d, *J*_1,2_ = 1.6 Hz, 1H, H-1'), 5.65 (dd, *J*_1,2_ = 1.6 Hz, *J*_2,3_ = 3.2 Hz, 1H, H-2'), 5.32 (t, *J*_3,4_ = *J*_4,5_ =9.6 Hz, 1H, H-4), 5.13 (d, *J*_1,2_ = 1.6 Hz, 1H, H-1'), 4.73–4.69 (m, 1H, H-3), 4.50–4.43 (m, 2H), 4.27–4.20 (m, 2H), 3.99–3.91 (m, 2H), 3.64 (d, 1H, *J* 9.5 Hz, O*H*), 3.36–3.30 (m, 2H, H-6, O*H*), 2.17 (s, 3H, COC*H*_3_). ^13^C-NMR (75 MHz, CDCl_3_): δ 171.3 (*C*OCH_3_), 166.1, 165.6, 165.5, 165.4 (4 × *C*OPh), 160.6(*C*NO_2_), 142.9, 133.5, 133.4, 133.2, 133.1, 129.8, 129.7, 129.6, 129.1, 129.0, 128.8, 128.6, 128.5, 128.4, 128.2, 126.0, 116.2, 97.73, 97.70 (2 × C-1), 77.2, 70.4, 70.3, 70.1, 69.9, 69.8, 69.3, 69.1, 66.8, 66.6, 62.8, 20.8 (CO*C*H_3_). HRMS for C_48_H_47_N_2_O_18_ (M+NH_4_)^+^ 939.28184. Found: 939.28088.

*4-Nitrophenyl 2,3,4,6-tetra-O-benzoyl-α-d-mannopyranosyl-(1→6)-[2,3,4,6-tetra-O-benzoyl-α-d- mannopyranosyl-(1→3)]-4-O-acetyl-α-d-mannopyranoside* (**8**): Glycosylation between disaccharide acceptor **7** (2.8 g, 3 mmol) and monosaccharide donor **4** (2.4 g, 3.1 mmol) was accomplished by following the same procedure as described above for the preparation of ditrasaccharide **5**. After purification, trisaccharide **8** (3.9 g, 86%) was afforded as a white foamy solid. 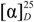 –27.2° (*c* 0.3 CHCl_3_). ^1^H-NMR (300 MHz, CDCl_3_): δ 8.30–7.16 (m, 44H, Bz-*H*, Ar-*H*), 6.09 (t, *J*_3,4_ = *J*_4,5_ = 10.1 Hz, 2H, H-4', H-4''), 5.93 (dd, *J*_2,3_ = 3.3 Hz, *J*_3,4_ = 10.1 Hz, 1H, H-3'), 5.80 (dd, *J*_2,3_ = 3.3 Hz, *J*_3,4_ = 10.1 Hz, 1H, H-3''), 5.63–5.51 (m, 4H, H-2', H-2'', H-1', H-4), 5.42 (s, 1H, H-1''), 5.10 (d, *J*_1,2_ = 1.6 Hz, 1H, H-1), 4.77–4.63 (m, 4H), 4.52–4.44 (m, 3H), 4.35 (dd, *J*_2,3_ = 3.1 Hz, *J*_3,4_ = 9.5 Hz, 1H, H-3), 4.0 (d, *J* = 8.8 Hz, 2H, 2H-6), 3.61 (d, *J* = 8.8 Hz, 1H, H-6), 3.06 (d, *J* = 4.9 Hz, 1H, O*H*), 2.27 (s, 3H, COC*H*_3_). ^13^C NMR (75 MHz, CDCl_3_): δ 170.3 (*C*OCH_3_), 166.2, 166.1, 165.6(2), 165.4, 165.3(2), 165.0 (8 × *C*OPh), 160.3 (*C*NO_2_), 143.0, 133.6, 133.4, 133.3, 133.0, 129.8, 129.75, 129.7, 129.6, 129.56, 129.50, 129.1, 129.0, 128.99, 128.91, 128.8, 128.7, 128.6, 128.5, 128.4, 128.3, 128.28, 128.22, 128.1, 125.9, 116.2, 99.6, 97.6, 97.5 (3 × C-1), 79.0, 77.2, 70.8, 70.6, 70.3, 69.8, 69.7, 69.6, 69.4, 69.0, 67.1, 67.0, 66.7, 63.4, 62.8, 20.7 (CO*C*H_3_). HRMS for C_82_H_69_NO_27_Na (M+Na)^+^ 1522.39508. Found: 1522.39463.

*4-Nitrophenyl 2,3,4,6-tetra-O-benzoyl-α-d-mannopyranosyl-(1→6)-[2,3,4,6-tetra-O-benzoyl-α-d-mannopyranosyl-(1→3)]-2,4-di-O-acetyl-α-d-mannopyranoside* (**9**): To a solution of **8** (105 mg, 0.07 mmol) in pyridine (2 mL) was added Ac_2_O (1 mL). The reaction mixture was stirred at rt for 12 h, at the end of which time TLC (petroleum ether–EtOAc 2:1) indicated that the reaction was complete. The reaction mixture was concentrated, and then the residue was purified by flash column chromatography on a silica gel column (petroleum ether–EtOAc 3:1) to give compound **9** (102 mg, 95%) as a yellow syrup. ^1^H-NMR (300 MHz, CDCl_3_): δ 8.30–7.16 (m, 44H, Bz-*H*, Ar-*H*), 6.16, 6.11 (2t, *J*_3,4_ = *J*_4,5_ =9.9 Hz, 2H, H-4', H-4''), 5.83 (dd, *J*_2,3_ = 3.3 Hz, *J*_3,4_ =9.9 Hz, 1H, H-3'), 5.80 (dd, *J*_2,3 _ = 3.3 Hz, *J*_3,4_ = 9.9 Hz, 1H, H-3''), 5.70 (d, *J*_1,2_ = 1.6 Hz, 1H, H-1'), 5.65 (dd, *J*_1,2_ =1.6 Hz, *J*_2,3_ = 3.3 Hz, 1H, H-2'), 5.58 (dd, *J*_1,2_ = 1.6 Hz, *J*_2,3_ = 3.3 Hz, 1H, H-2'') 5.53 (m, 2H, H-2'', H-4), 5.44 (d, *J*_1,2_ = 1.6 Hz, 1H, H-1''), 5.07 (d, *J*_1,2_ = 1.5 Hz, 1H, H-1) 4.71–4.48 (m, 7H), 4.05–4.00 (m, 2H), 3.61 (d, *J* = 8.8 Hz, 1H, H-6), 2.36 (s, 3H, COC*H*_3_), 2.28 (s, 3H, COC*H*_3_). ^13^C-NMR (75 MHz, CDCl_3_): δ 170.6, 170.0 (2 × *C*OCH_3_), 166.2, 166.1, 165.6, 165.5, 165.4, 165.3, 165.2(2) (8 × *C*OPh), 160.0 (*C*NO_2_), 143.3, 133.6, 133.5, 133.4, 133.2, 133.1, 132.9, 129.8, 129.7, 129.5, 129.2, 129.1, 129.0, 128.98, 128.95, 128.6, 128.57, 128.52, 128.4, 128.36, 128.31, 128.2, 126.0, 116.4, 99.1, 97.6, 95.7 (3 × C-1), 77.2, 74.2, 70.8, 70.7, 70.3, 70.2, 69.9, 69.7, 69.2, 69.1, 67.7, 66.8, 66.7, 66.6, 62.9, 62.8, 20.7, 20.8 (2 × CO*C*H_3_). HRMS for C_84_H_71_NO_28_Na (M+Na)^+^ 1564.40562. Found: 1564.40820.

*4-Nitrophenyl α-d-mannopyranosyl-(1→6)-[α-d-mannopyranosyl-(1→3)]-α-d-mannopyranoside* (**10**): Compound **8** (3.2 g, 2.1 mmol) was dissolved in satd NH_3_-MeOH (120 mL). After 120 h at rt, the reaction mixture was concentrated to a total volume of about 5 mL, then warm acetone (50 mL, 50 °C) was added to the mixture under vigorous stirring, and a white solid precipitate from the solution, after kept at 0 °C for 24 h, target compound **10** was collected after filtration (973 mg, 73%) as white solid. 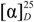 +54.2° (*c* 0.1 DMSO). ^1^H-NMR (300 MHz, D_2_O): δ 8.20 (d, *J* = 9.2 Hz, 2H, Ar-*H*), 7.20 (d, *J* = 9.2 Hz, 2H, Ar-*H*), 5.69 (s, 1H, H-1), 5.13 (s, 1H, *H*-1'), 4.30 (m, 1H, *H*-2), 4.11 (dd, *J*_2,3_ = 3.2 Hz, *J*_3,4_ = 9.3 Hz, 1H, *H*-3), 4.04 (m, 1H, *H*-2'), 3.90–3.80 (m, 5H), 3.78–3.74 (m, 3H), 3.68 (m, 2H), 3.64–3.61 (m, 2H), 3.57–3.54 (m, 2H). ^13^C-NMR (75 MHz, D_2_O): δ 160.6 (*C*NO_2_), 142.4, 126.1(2), 116.9(2) (4 × Ar-*C*), 102.4, 98.9, 97.6 (3 × C-1), 77.9, 73.2, 72.6, 71.9, 70.6, 70.4, 70.1, 69.9, 69.2, 66.8, 66.7, 65.8. 65.2, 61.0, 60.9. HRMS for C_24_H_35_NO_18_Na (M+Na)^+^ 648.1752. Found: 648.1754.

## 4. Conclusions

In summary, a highly efficient and regioselective procedure with a simple work-up which is applicable on a large-scale has been developed for the direct synthesis of the 2,3-*O*-isopropylidene-α-d-mannopyranoside derivatives from α-d-mannopyranosides. The high selectivity, high isolated yields, and widely application of the reaction highlight the usefulness of the method as a practical orthogonal protecting strategy in carbohydrate synthesis. The synthetic utility of this novel process was demonstrated through the successfully synthesis of a 3,6-branched α-d-mannosyl trisaccharide. It is noteworthy that overall yield of the whole synthesis is 50.4% from the corresponding mannoside, and the procedure is suitable for large scale preparation of the target trisaccharide.

## Supplementary Materials

Supplementary materials can be accessed at: http://www.mdpi.com/1420-3049/19/5/6683/s1.
