# Supplementary material for: Direct 2,3-O-Isopropylidenation of α-d-Mannopyranosides and the Preparation of 3,6-Branched Mannose Trisaccharides
Source: Molecules. 2014 May 22;19(5):6683–93. doi: 10.3390/molecules19056683 (PMC6270901; doi:10.3390/molecules19056683)
Supplement: Supplementary file 1 [file molecules-19-06683-s001.pdf]

Supplementary

^{1}H-NMR spectrum of compound **2a** S2
^{1}H-NMR spectrum of compound **3a** S2
^{13}C-NMR spectrum of compound **3a** S3
^{1}H-NMR spectrum of compound **3b** S3
^{1}H-NMR spectrum of compound **3c** S4
^{1}H-NMR spectrum of compound **3d** S4
^{1}H-NMR spectrum of compound **3e** S5
^{1}H-NMR spectrum of compound **3f** S5
^{1}H-NMR spectrum of compound **3g** S6
^{1}H-NMR spectrum of compound **4** S6
^{1}H-NMR spectrum of compound **5** S7
^{13}C-NMR spectrum of compound **5** S7
^{1}H-NMR spectrum of compound **6** S8
^{13}C-NMR spectrum of compound **6** S8
^{1}H-NMR spectrum of compound **7** S9
^{13}C-NMR spectrum of compound **7** S9
^{1}H-NMR spectrum of compound **8** S10
^{13}C-NMR spectrum of compound **8** S10
^{1}H-NMR spectrum of compound **9** S11
^{13}C-NMR spectrum of compound **9** S11
^{1}H-NMR spectrum of compound **10** S12
^{13}C-NMR spectrum of compound **10** S12
HRMS spectrum of compound **10** S13

^{1}H-NMR spectrum of compound **2a**

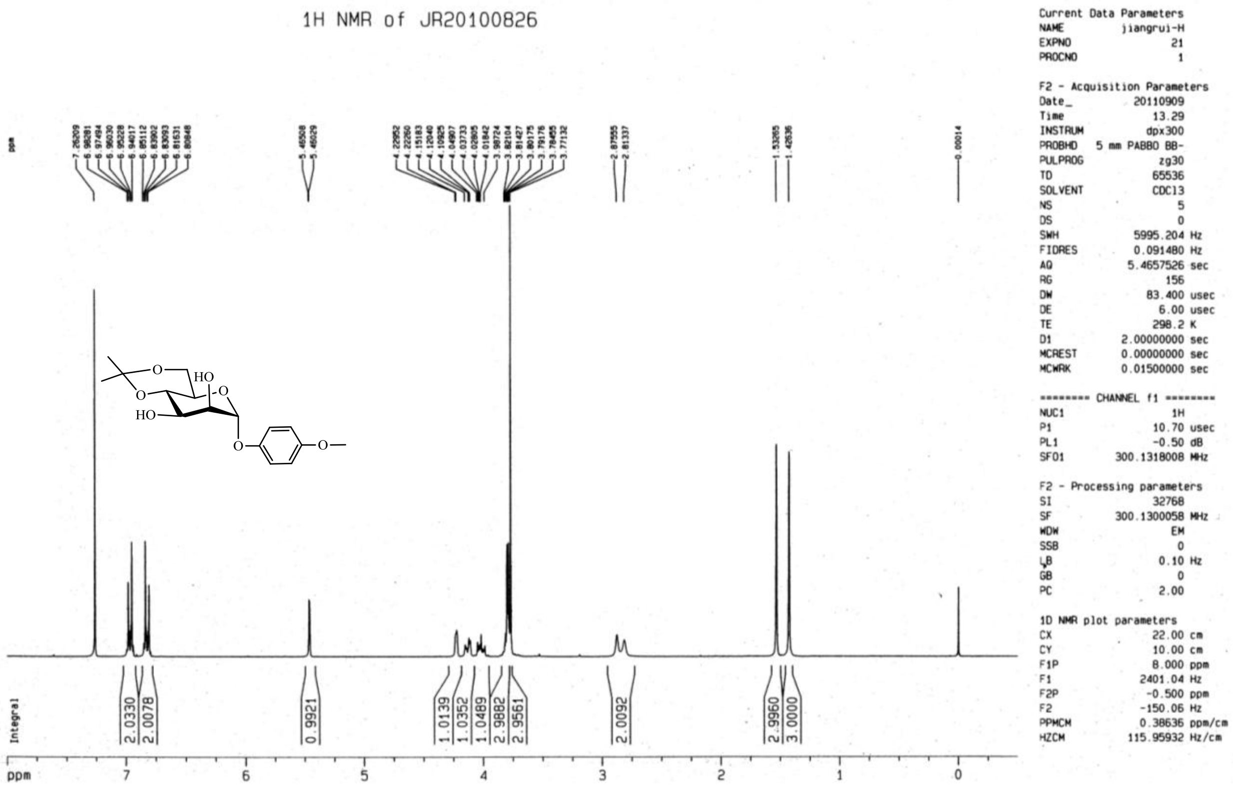

^{1}H-NMR spectrum of compound **3a**

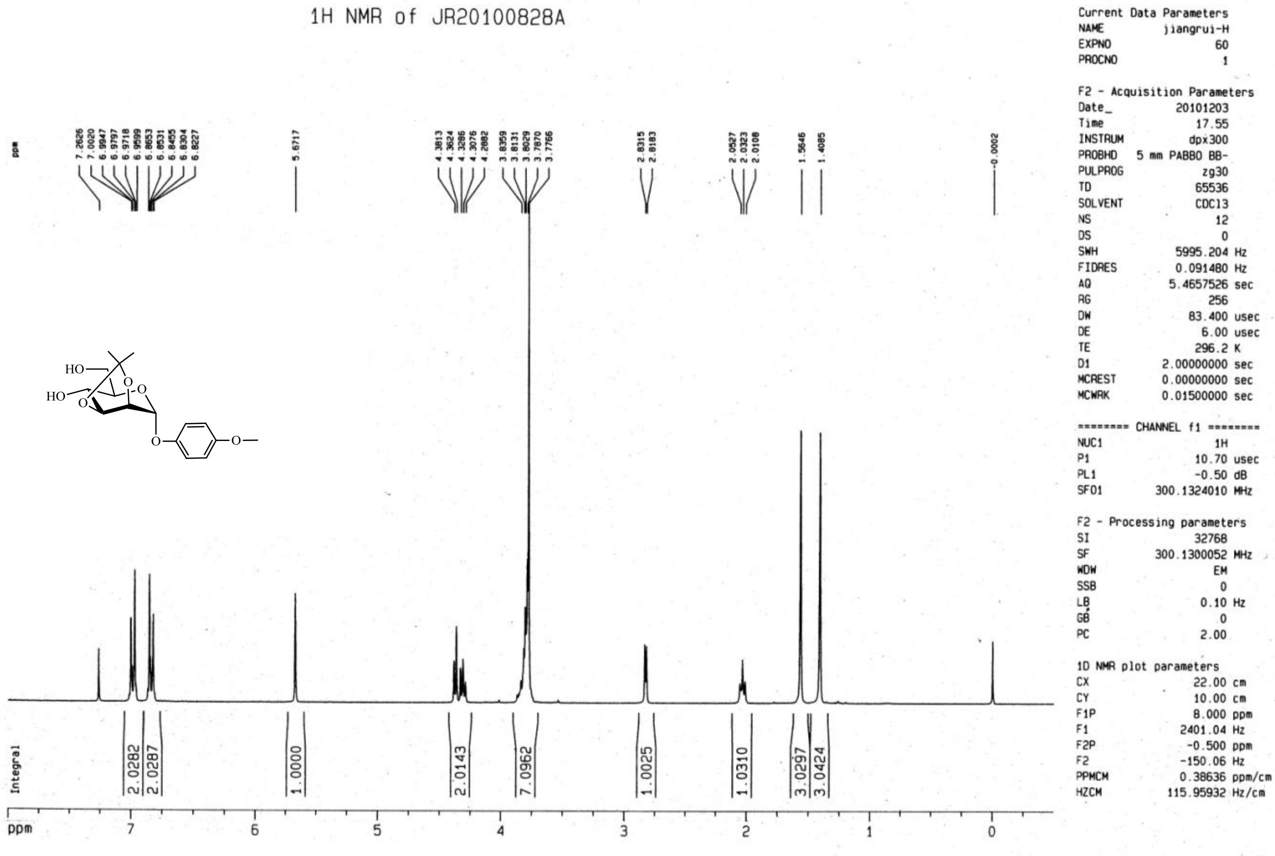

^{13}C-NMR spectrum of compound **3a**

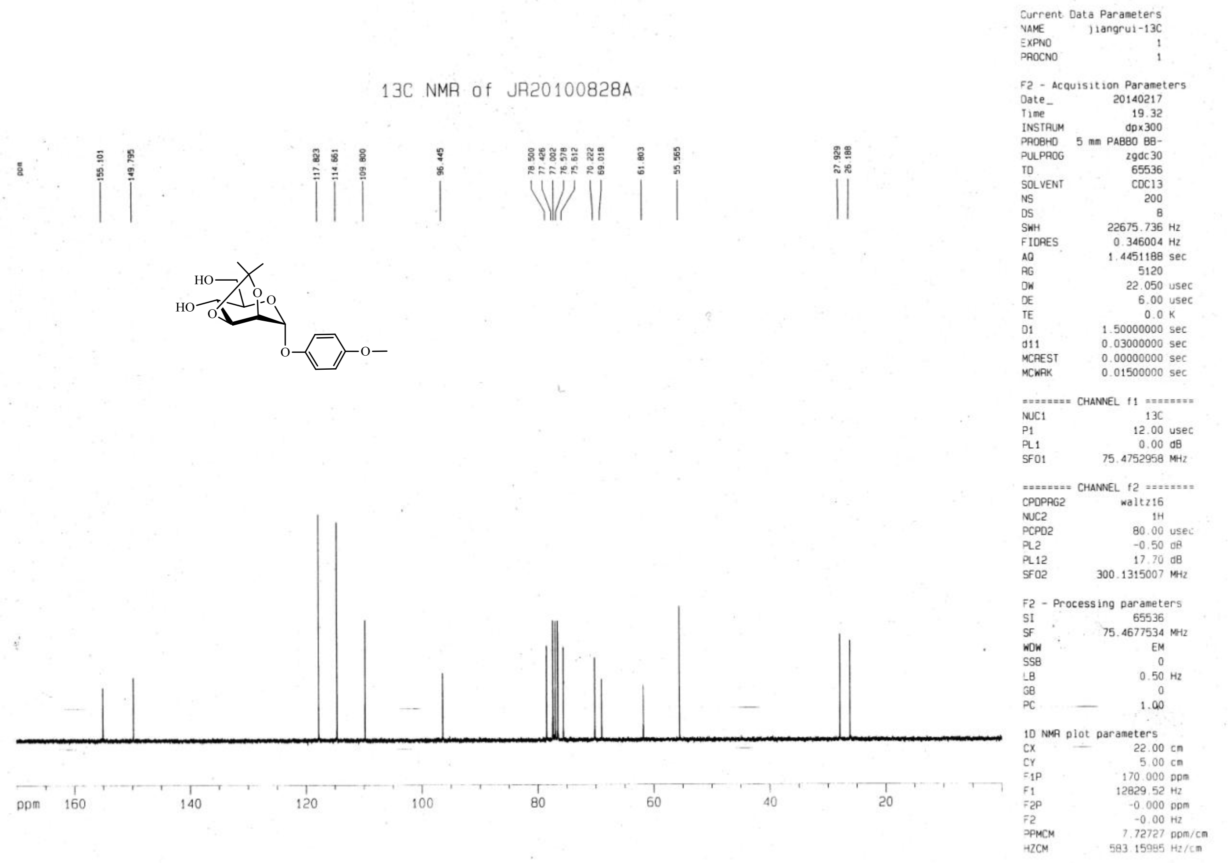

^{1}H-NMR spectrum of compound **3b**

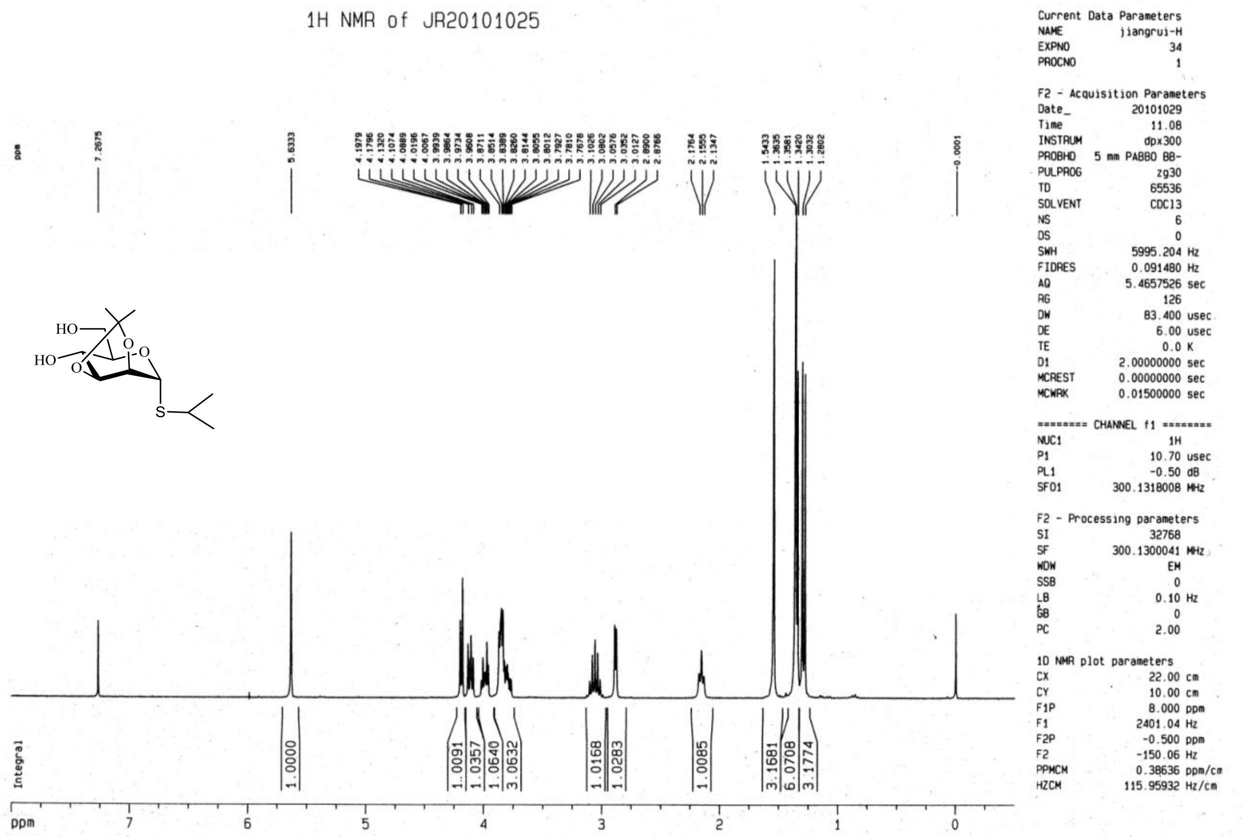

^{1}H-NMR spectrum of compound **3c**

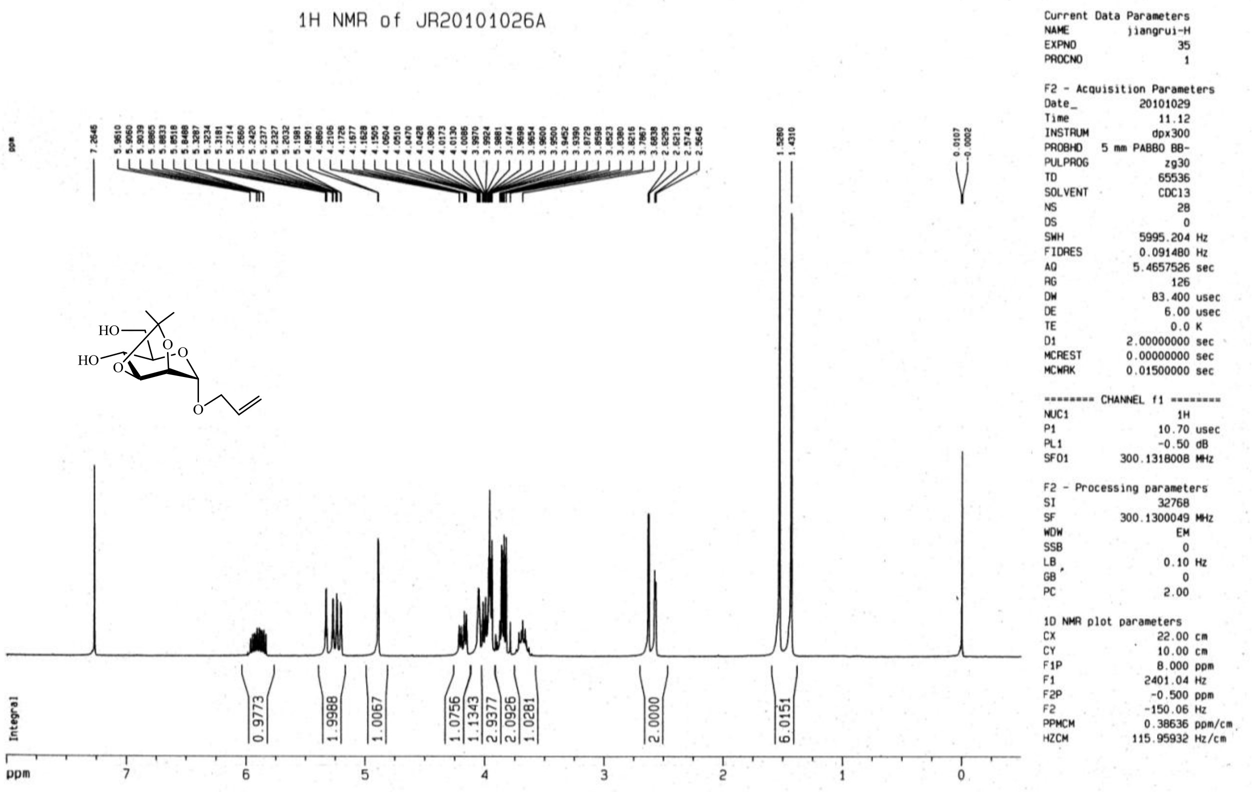

^{1}H-NMR spectrum of compound **3d**

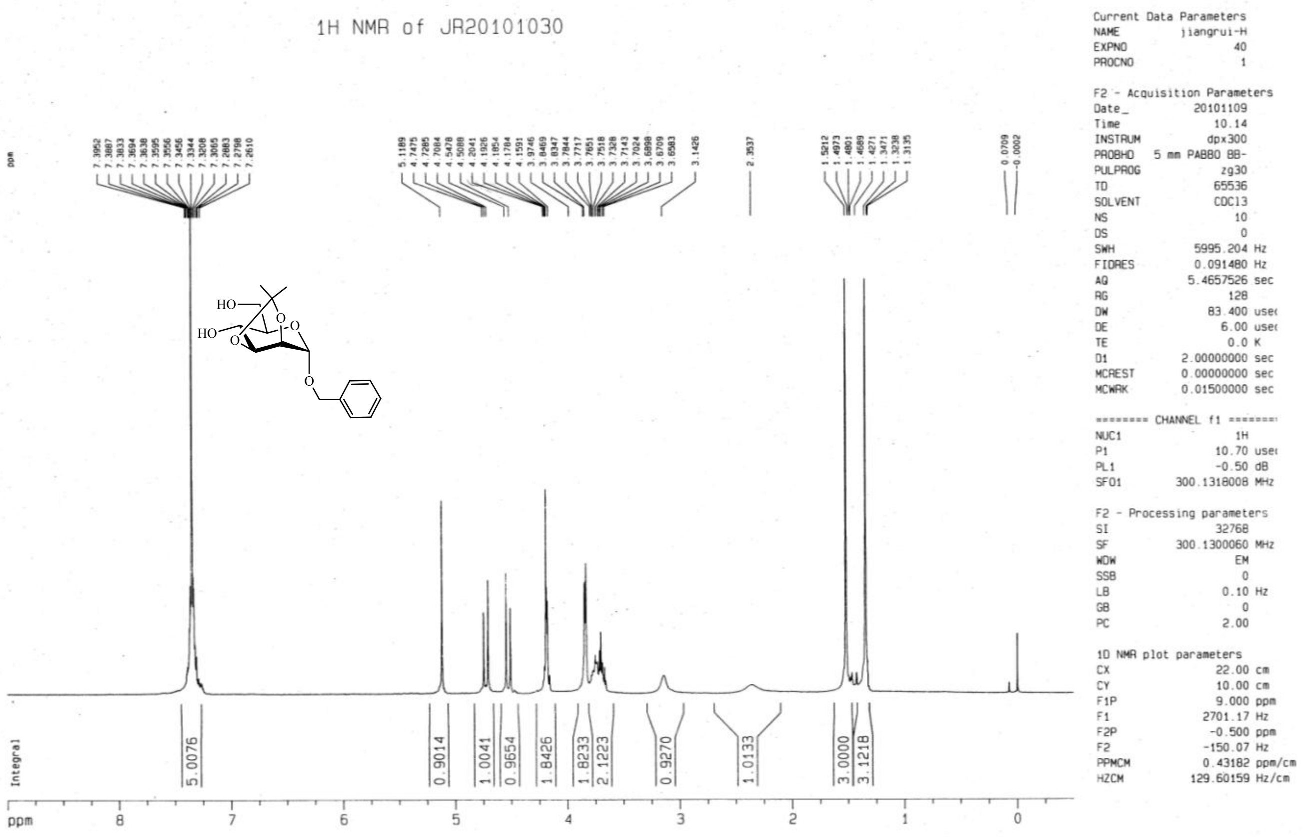

^{1}H-NMR spectrum of compound **3e**

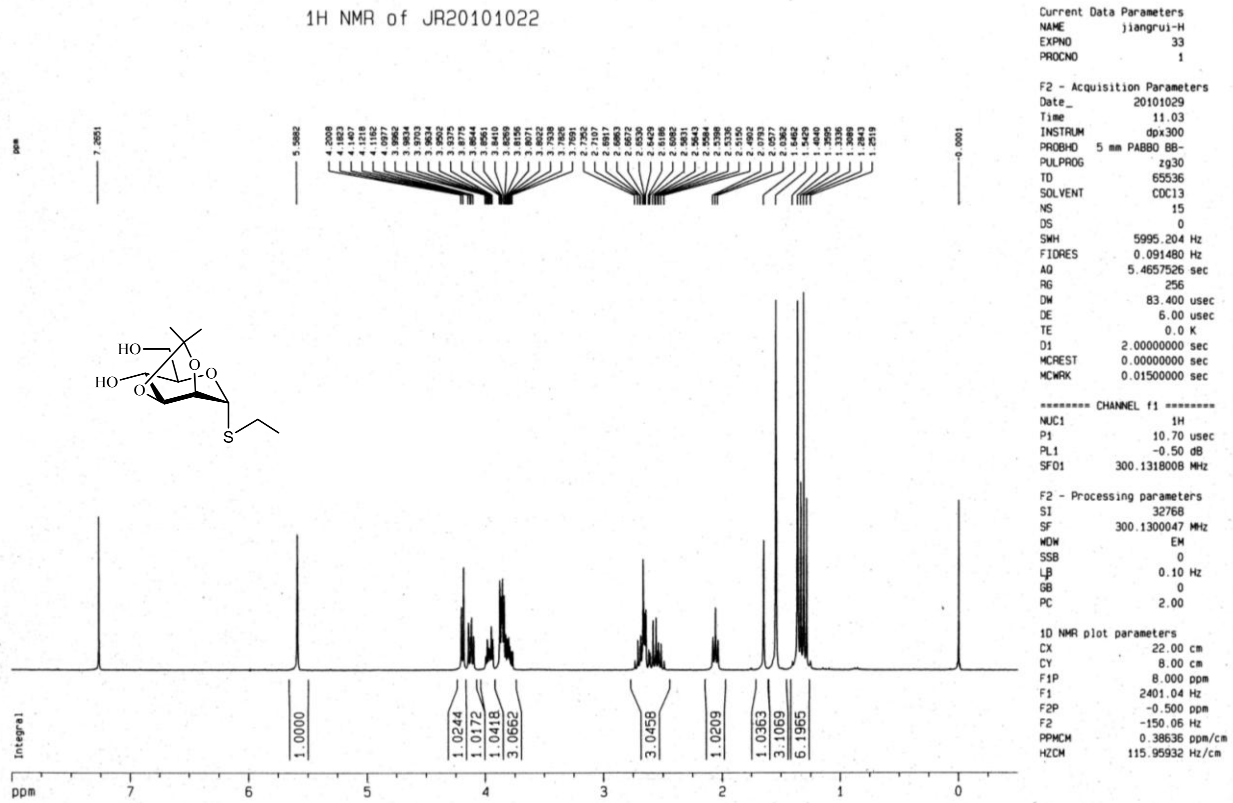

^{1}H-NMR spectrum of compound **3f**

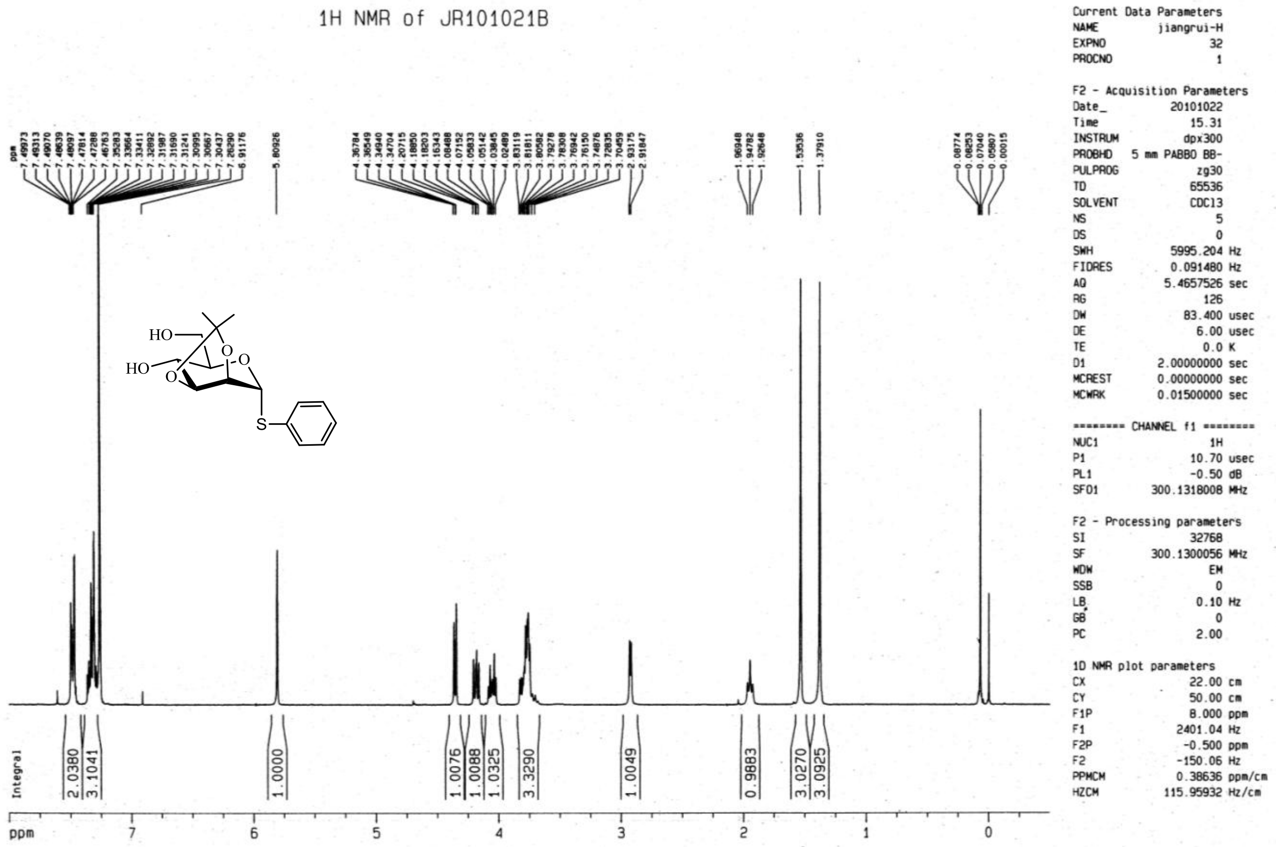

^{1}H-NMR spectrum of compound **3g**

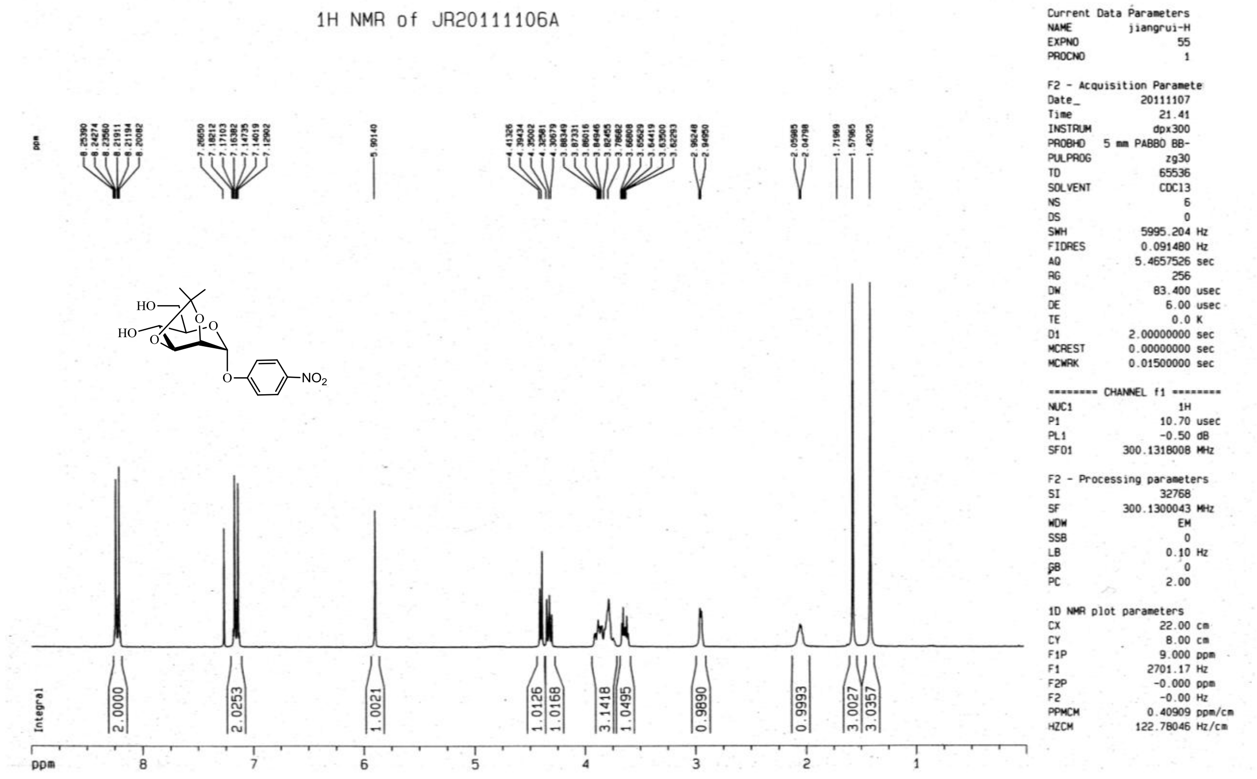

^{1}H-NMR spectrum of compound **4**

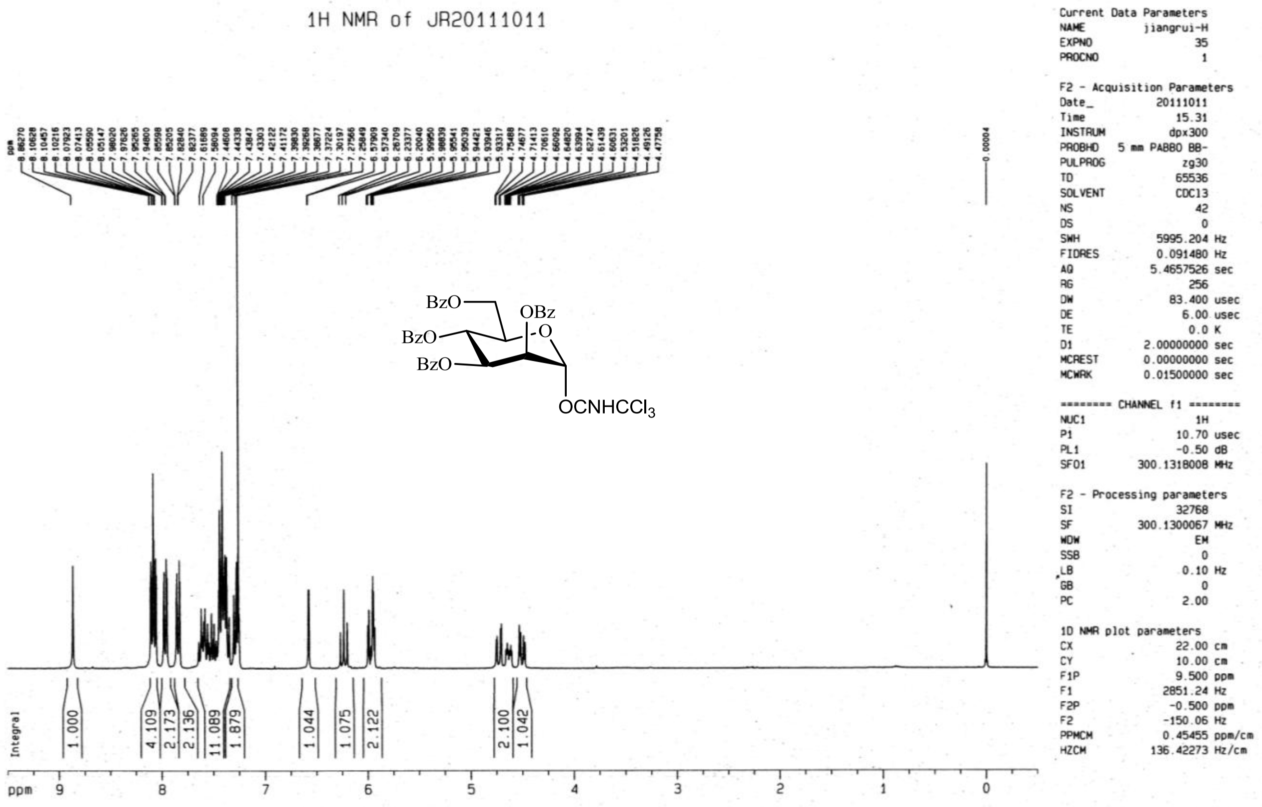

^{1}H-NMR spectrum of compound **5**

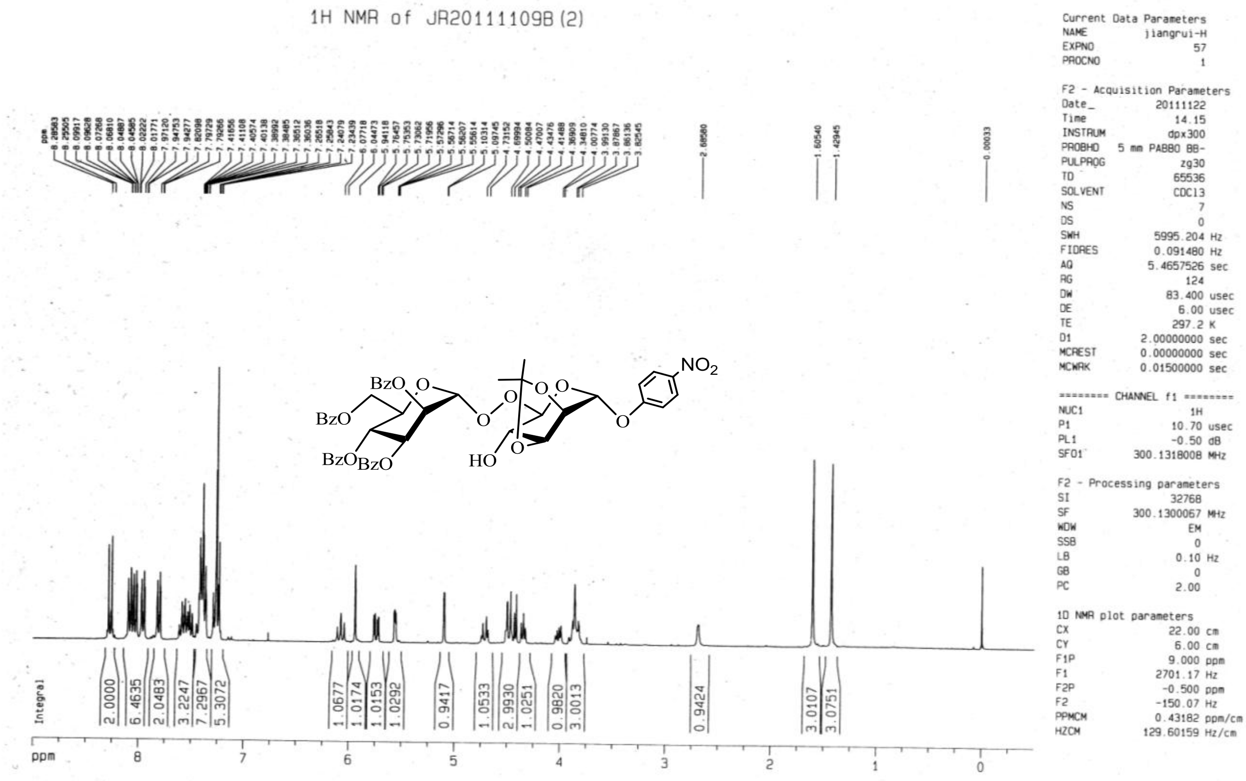

^{13}C-NMR spectrum of compound **5**

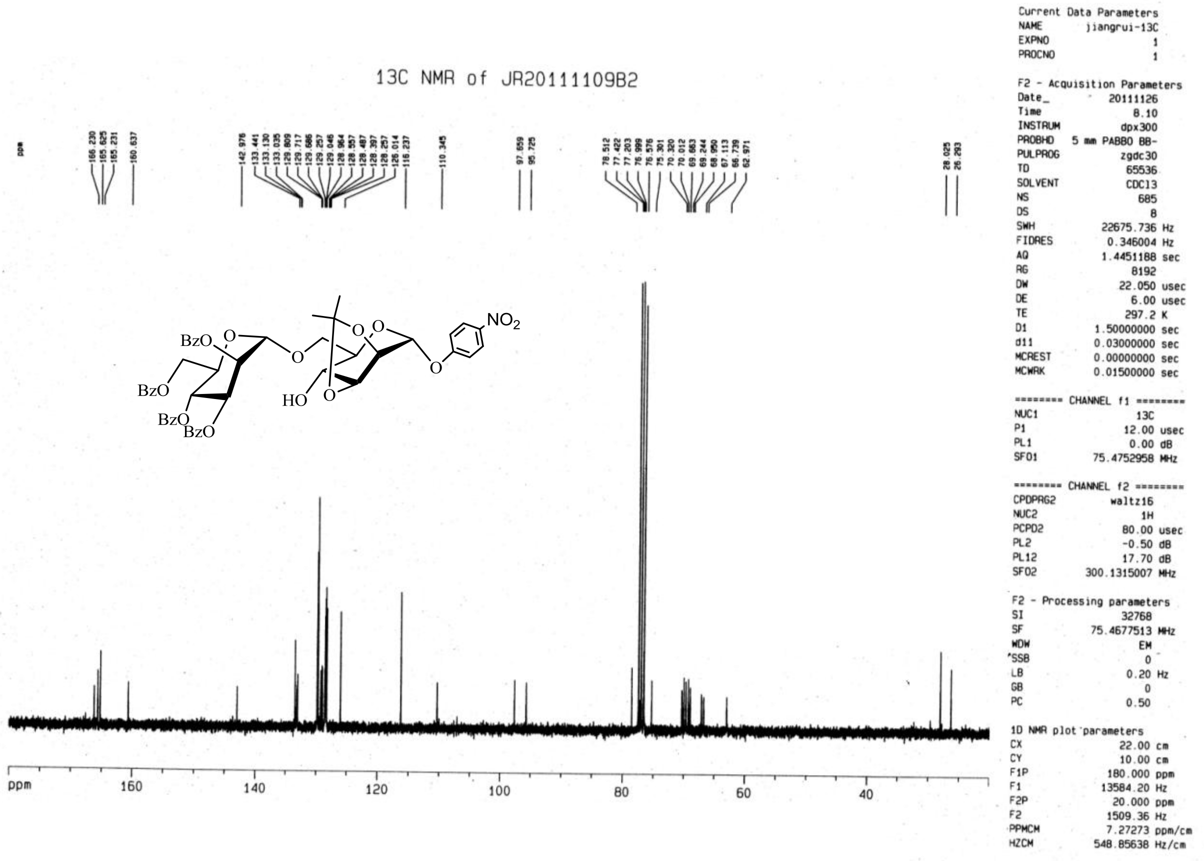

^{1}H-NMR spectrum of compound **6**

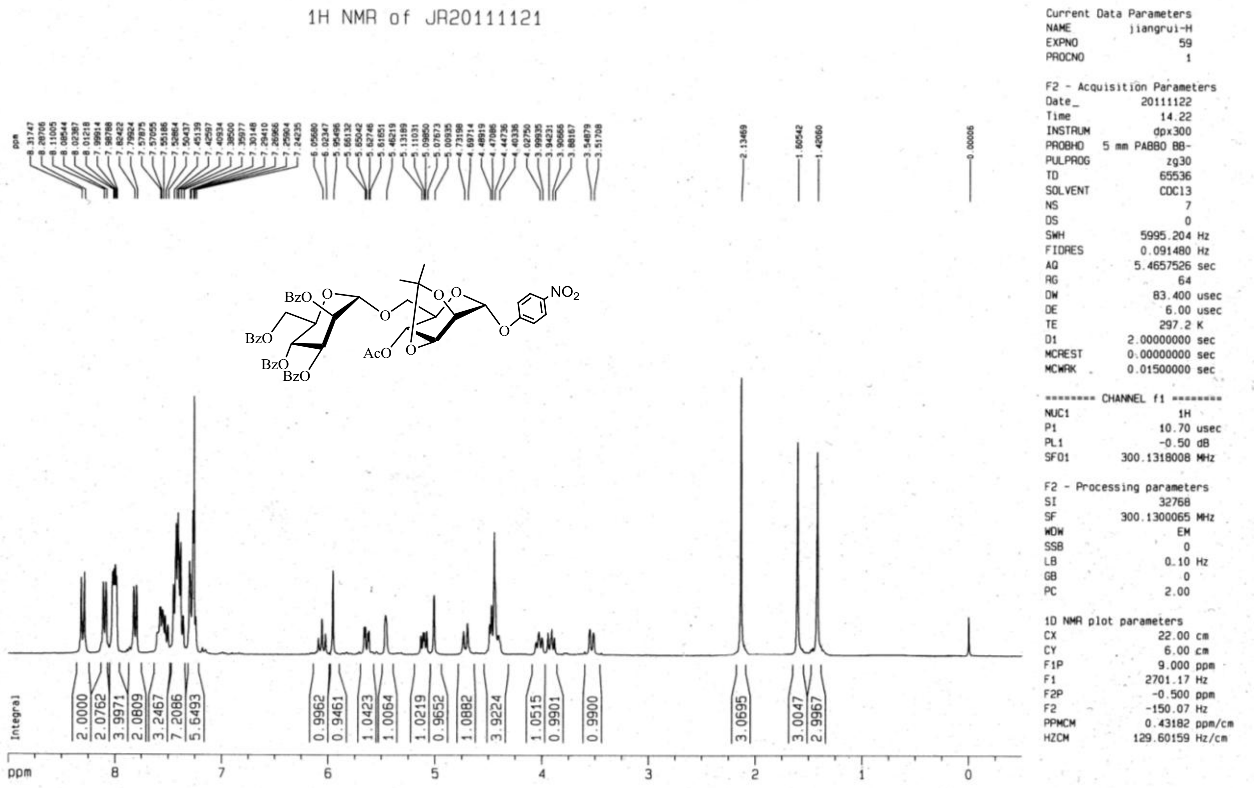

^{13}C-NMR spectrum of compound **6**

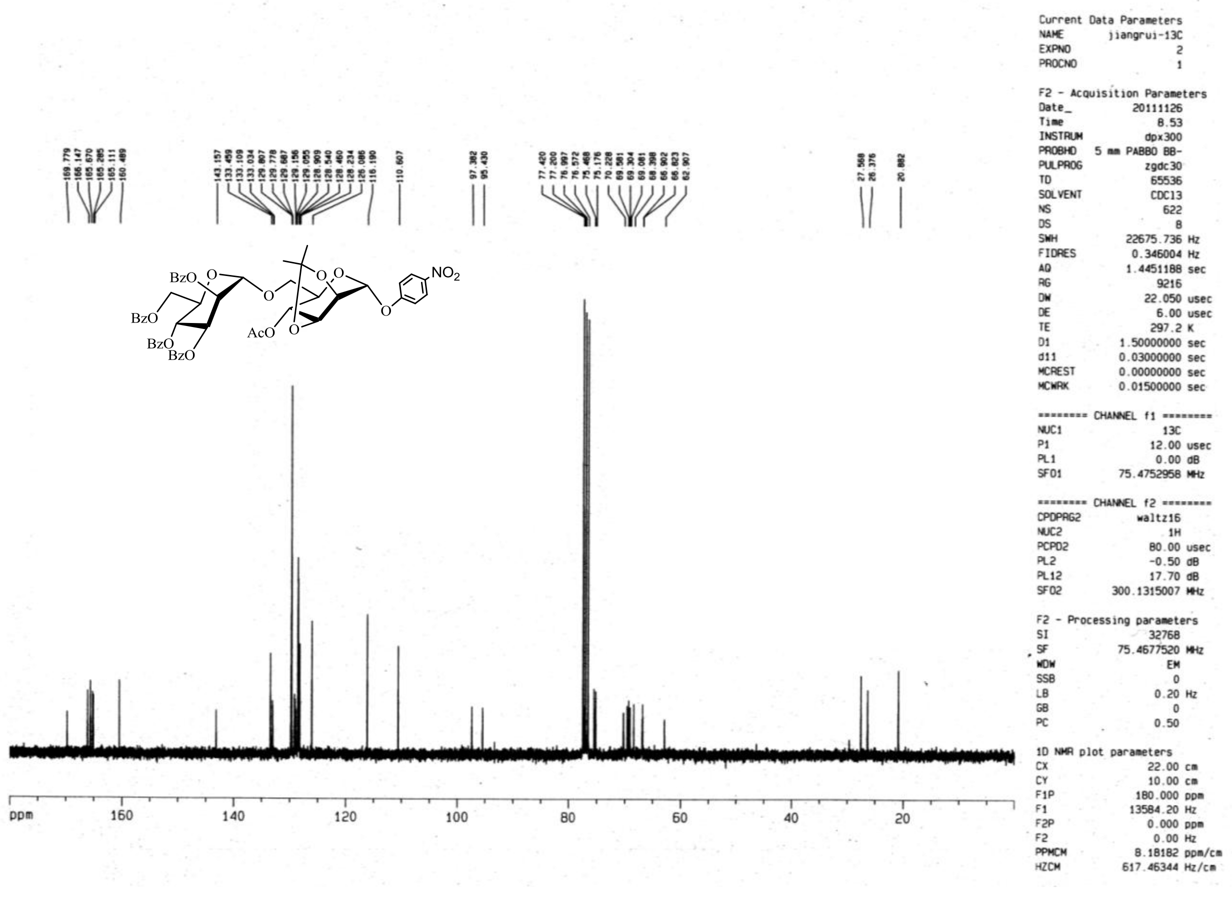

^{1}H-NMR spectrum of compound **7**

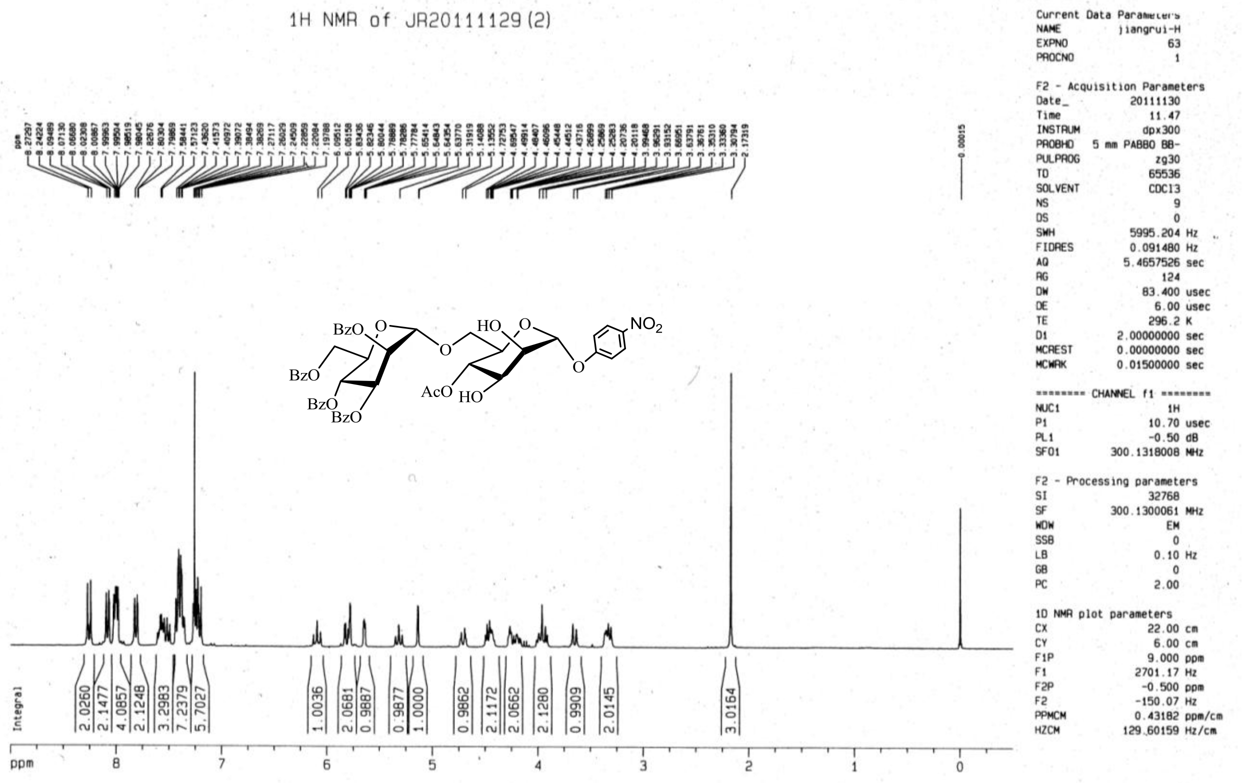

^{13}C-NMR spectrum of compound **7**

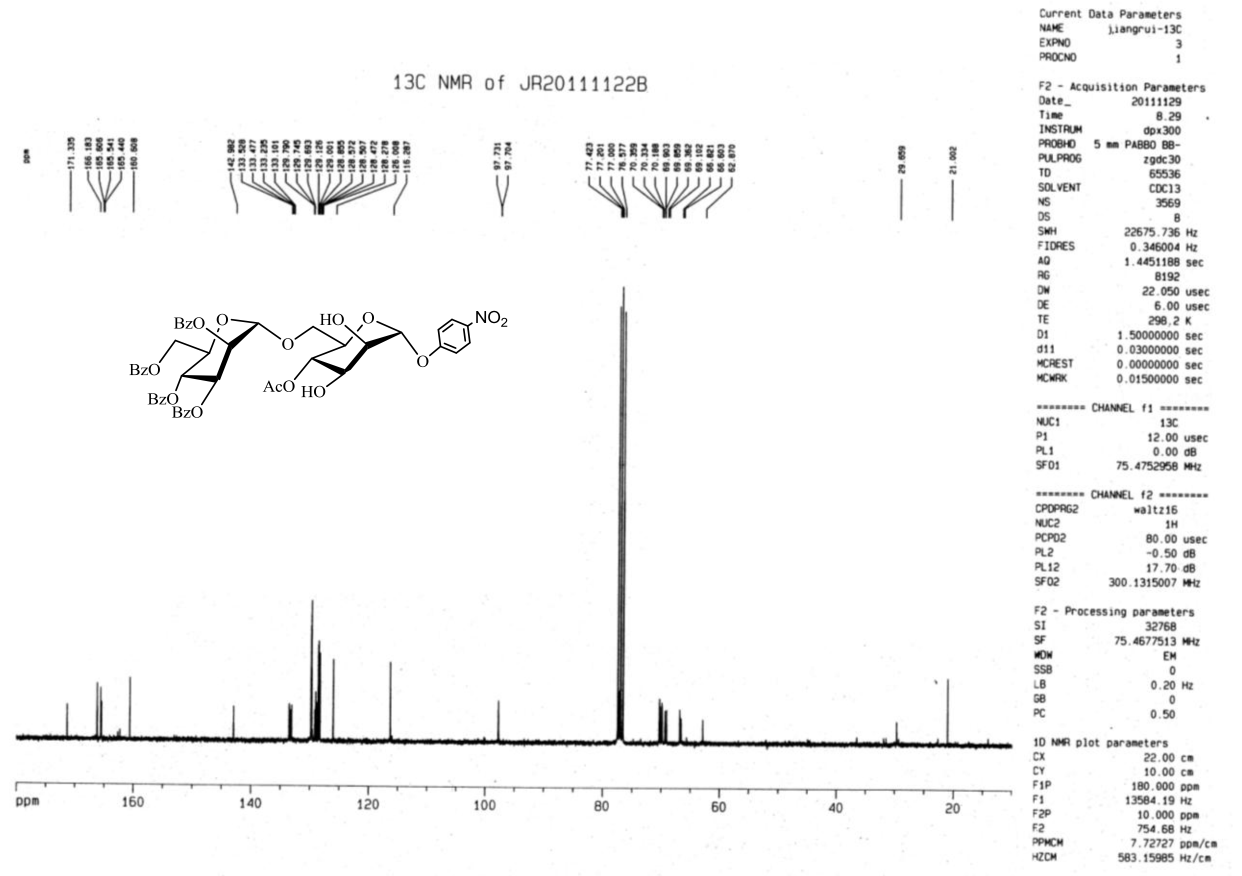

^{1}H-NMR spectrum of compound **8**

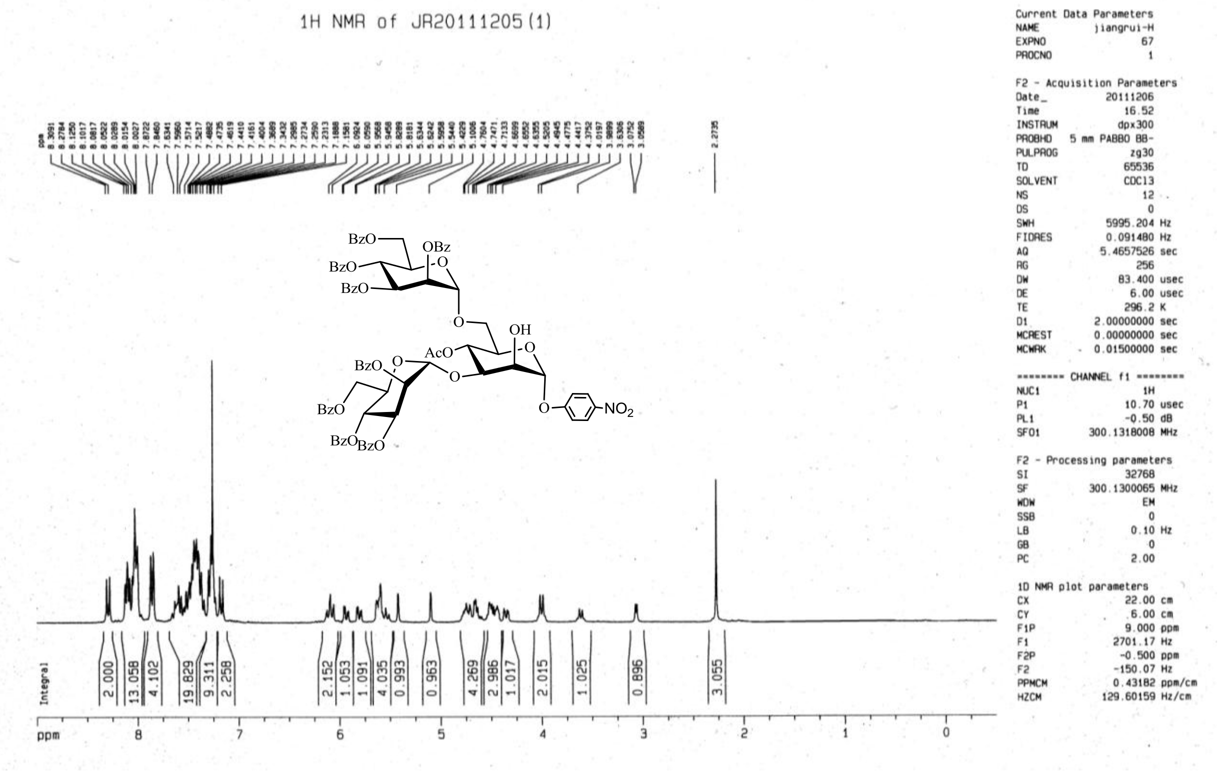

^{13}C-NMR spectrum of compound **8**

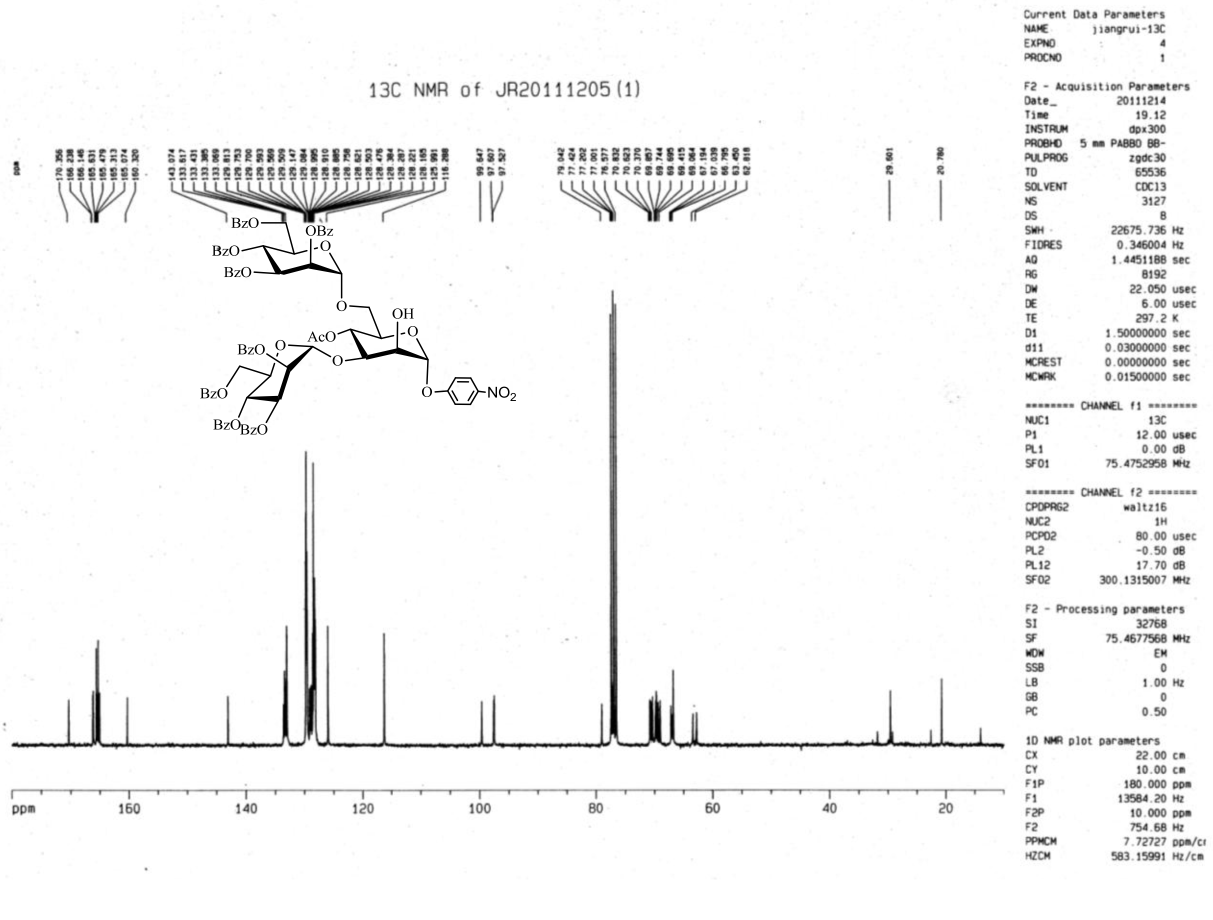

^{1}H-NMR spectrum of compound **9**

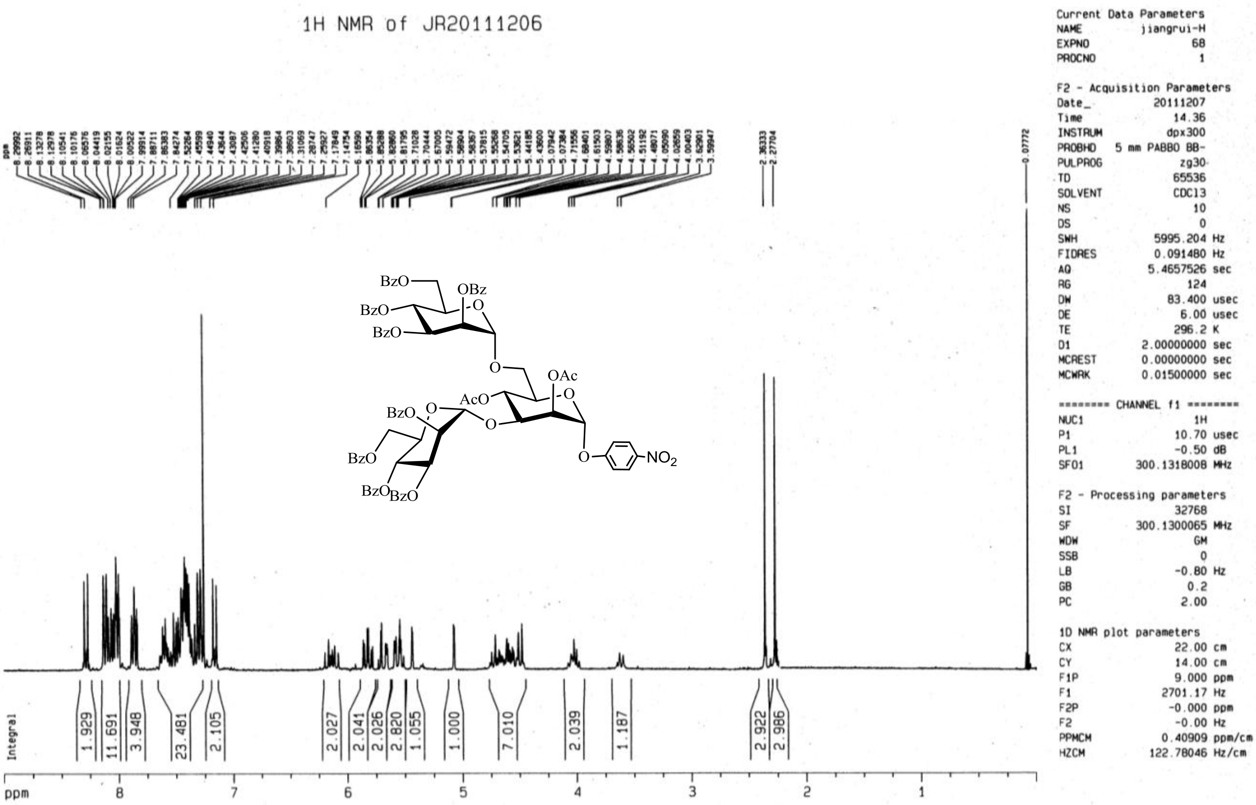

^{13}C-NMR spectrum of compound **9**

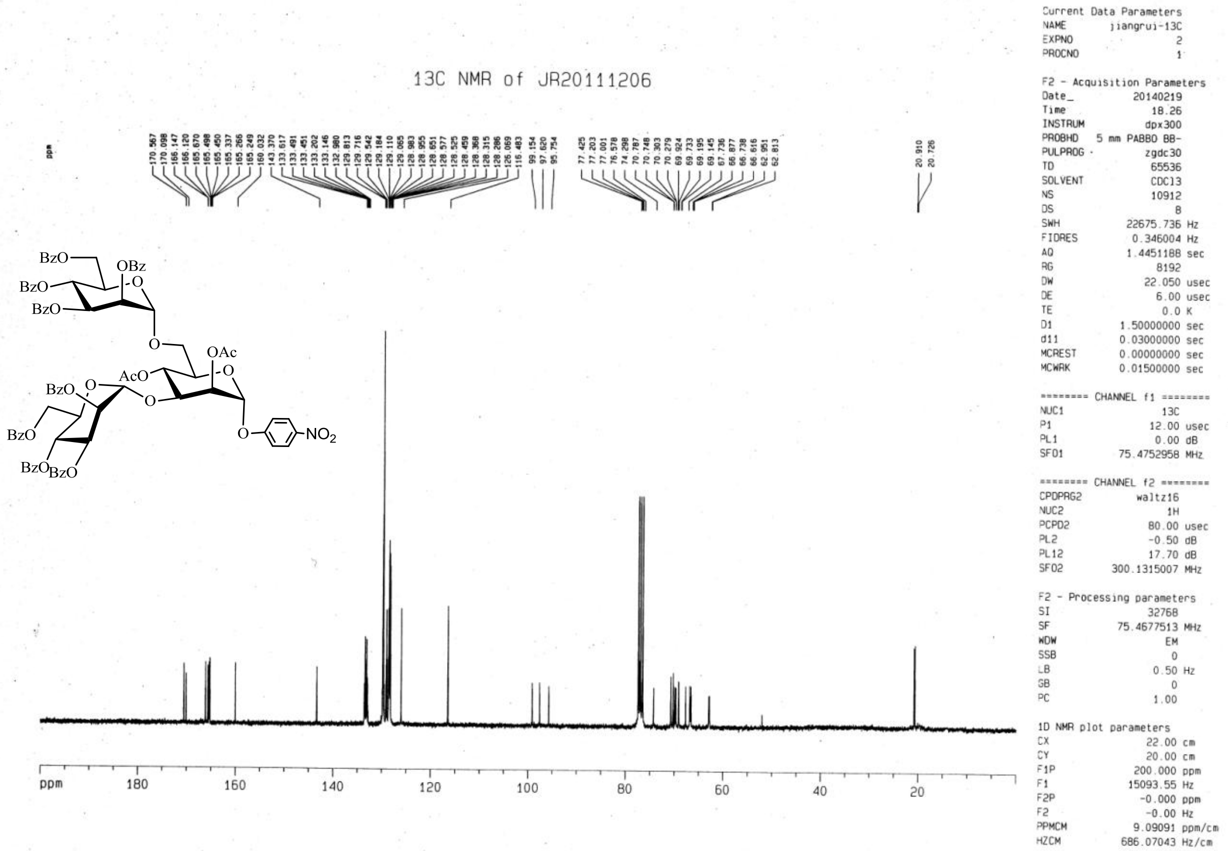

^{1}H-NMR spectrum of compound **10**

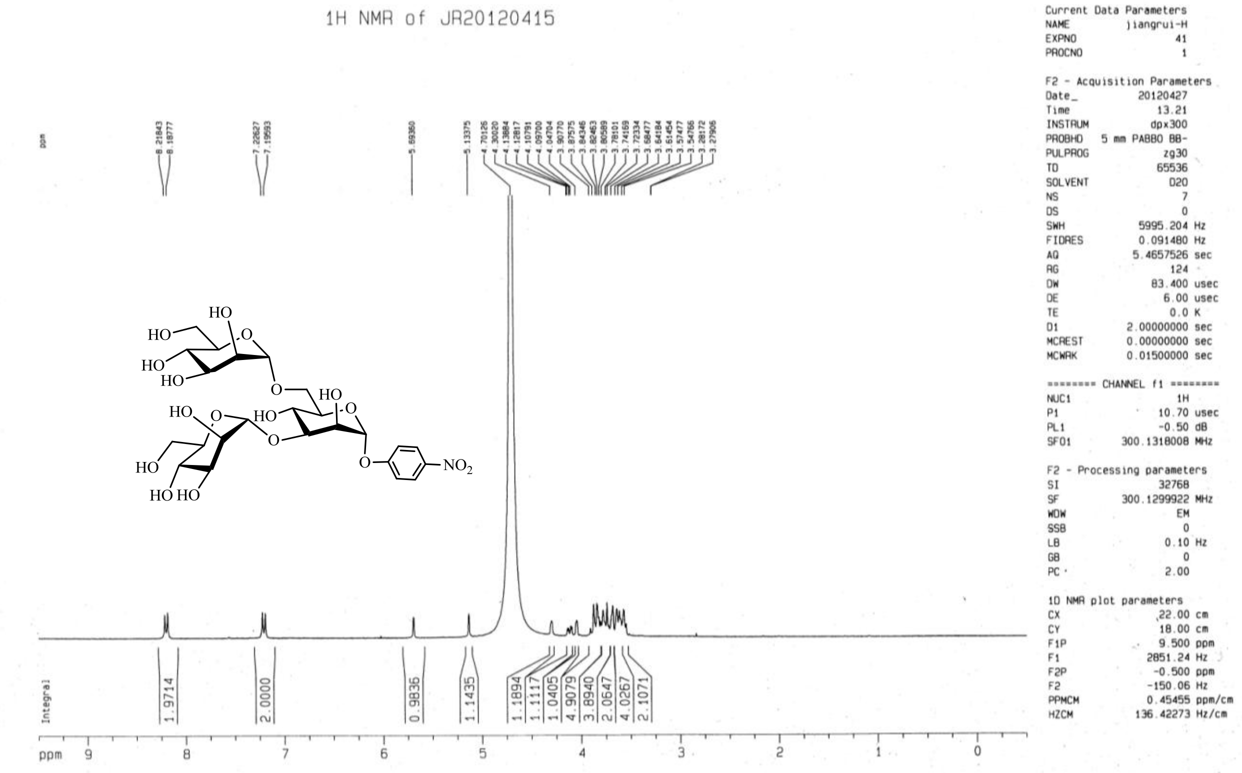

^{13}C-NMR spectrum of compound **10**

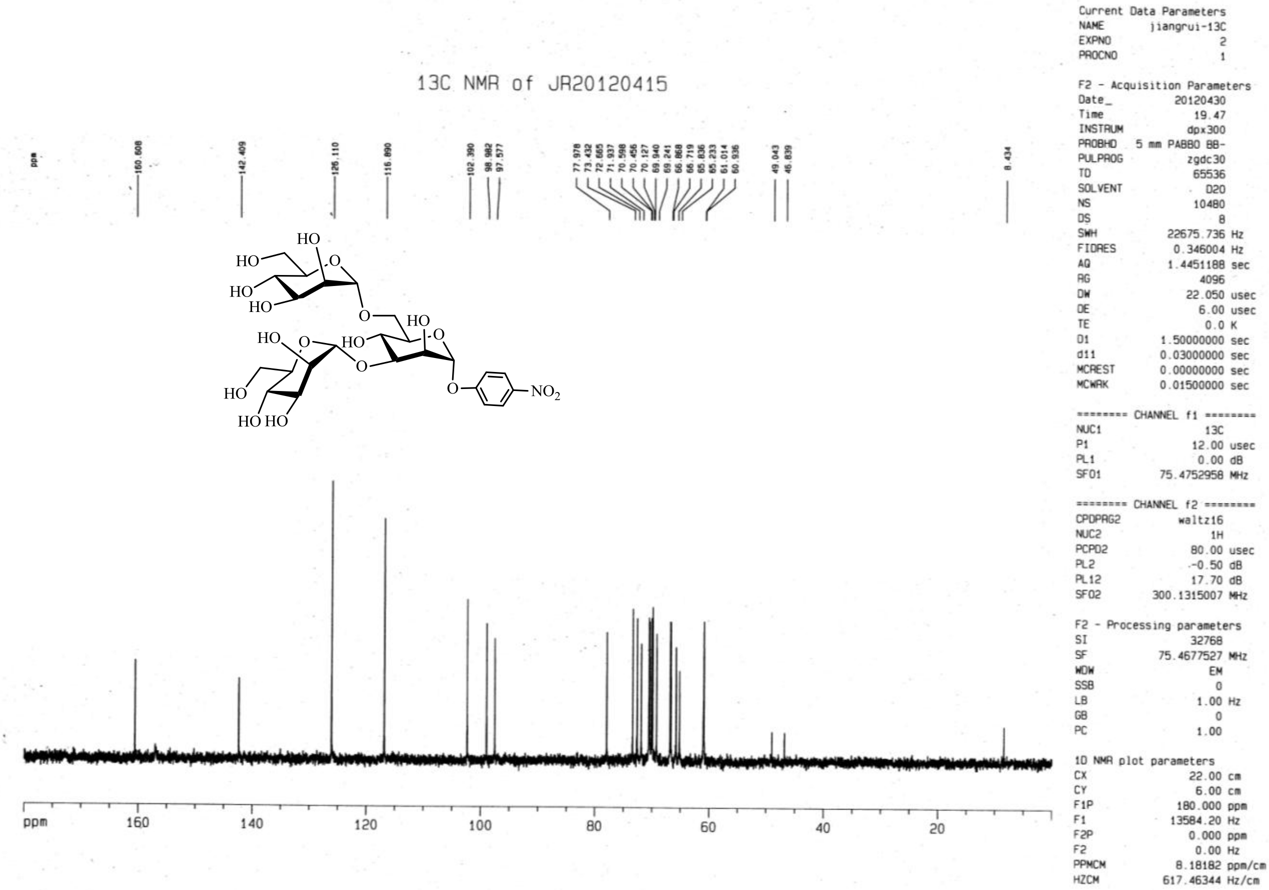

HRMS spectrum of compound **10**

Peking University Mass Spectrometry Sample Analysis Report

Analysis Info

Analysis Name	12050103_20120503_000001.d	Acquisition Date	5/3/2012 4:11:51 PM
Sample		Instrument	Bruker.Apex IV FTMS
Comment	ESI Positive	Operator	Peking University

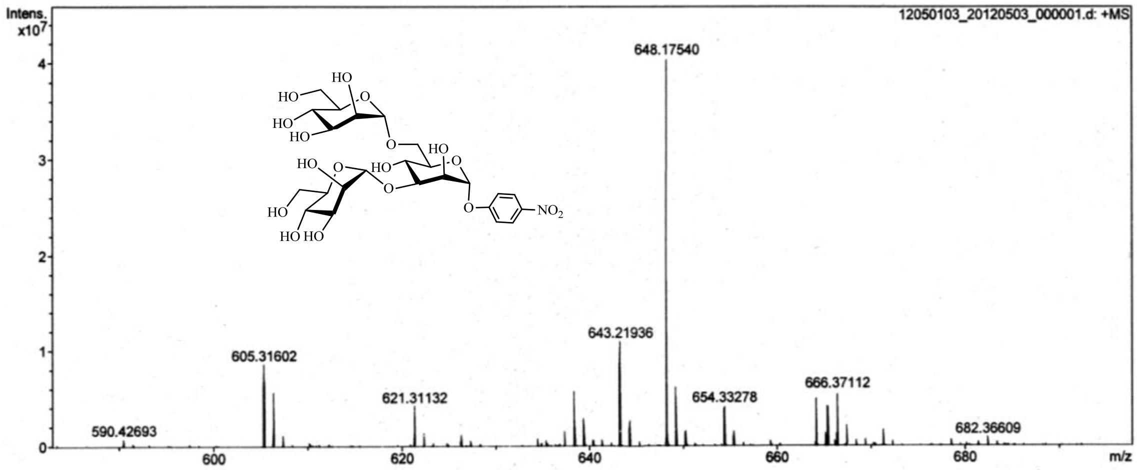

Meas. m/z	#	Formula	Score	m/z	err [mDa]	err [ppm]	mSigma	rdb	e⁻ Conf	N-Rule
643.21936	1	C 24 H 39 N 2 O 18	100.00	643.21924	-0.1	-0.2	3.7	6.5	even	ok
648.17540	1	C 24 H 35 N Na O 18	100.00	648.17463	-0.8	-1.2	3.7	7.5	even	ok

Bruker Compass DataAnalysis 4.0 printed: 5/3/2012 4:16:39 PM Page 1 of 1
